# Lipid normalization and stable isotope discrimination in Pacific walrus tissues

**DOI:** 10.1038/s41598-019-42095-z

**Published:** 2019-04-10

**Authors:** Casey T. Clark, Lara Horstmann, Nicole Misarti

**Affiliations:** 1 Water and Environmental Research Center, University of Alaska Fairbanks, 1764 Tanana Loop, Fairbanks, Alaska 99775-5860 USA; 2 College of Fisheries and Ocean Sciences, University of Alaska Fairbanks, 2150 Koyukuk Drive, Fairbanks, Alaska 99775-7220 USA

**Keywords:** Boreal ecology, Ecophysiology, Stable isotope analysis

## Abstract

Analysis of stable carbon and nitrogen isotope values (δ^13^C and δ^15^N) of animal tissues can provide important information about diet, physiology, and movements. Interpretation of δ^13^C and δ^15^N values, however, is influenced by factors such as sample lipid content, tissue-specific isotope discrimination, and tissue turnover rates, which are typically species- and tissue-specific. In this study, we generated lipid normalization models for δ^13^C and investigated the effects of chemical lipid extractions on δ^13^C and δ^15^N in Pacific walrus (*Odobenus rosmarus divergens*) muscle, liver, and skin. We also evaluated tissue-specific isotope discrimination in walrus muscle, liver, skin, and bone collagen. Mean δ^13^C_lipid-free_ of skin and bone collagen were similar, as were mean δ^15^N of muscle and liver. All other tissues differed significantly for both isotopes. Differences in δ^13^C_lipid-free_ and δ^15^N among tissues agreed with published estimates of marine mammal tissue-specific isotope discrimination factors, with the exception of skin. The results of this work will allow researchers to gain a clearer understanding of walrus diet and the structure of Arctic food webs, while also making it possible to directly compare the results of contemporary walrus isotope research with those of historic and paleoecological studies.

## Introduction

Stable isotope analysis is widely-used as a tool to study animal movements, diet, and food web structure^1–4^. Carbon and nitrogen stable isotope values (δ^13^C and δ^15^N) are particularly useful when evaluating animal diet and trophic position^3^. Consumer δ^13^C is driven mainly by the type of primary production at the base of the food web, as uptake of CO_2_ for use in photosynthesis involves discrimination of carbon isotopes^5^. Trophic discrimination of ^13^C is typically small (~1‰ per trophic level), but accounts for some variation in stable carbon isotope values^1,3^. In contrast, variability in δ^15^N is strongly influenced by trophic discrimination, which results in an increase of ~3.0‰ per trophic level^2,3,6^. By interpreting δ^13^C and δ^15^N values from animal tissues in the context of these sources of isotopic discrimination, information can be inferred about diet, trophic position, and the flow of energy through biological systems.

Additional processes that result in discrimination of ^13^C and ^15^N can complicate reconstructions of animal diet and trophic position. For example, discrimination against the heavier ^13^C during lipid synthesis results in differences of ~6–8‰ between lipids and proteins^7,8^. Lipid content therefore affects tissue δ^13^C and may lead to observed variability in δ^13^C among animals resulting from differences in lipid storage, rather than diet. Because lipids are not always homogenously distributed within a tissue^9^, lipid content can be an important source of isotopic variability even among multiple samples of a single tissue from one individual^10^. Furthermore, δ^13^C varies among fatty acids^11,12^, thus the δ^13^C of lipids depends on their fatty acid composition and uneven distribution of different types of lipids within a tissue may lead to heterogeneity in δ^13^C.

Chemical lipid extraction is one way to account for differences in sample lipid content. In this process, lipids are typically removed using polar organic solvents (often a mixture of chloroform and methanol^13,14^) and stable isotope values of the lipid-free sample are measured. This process allows for direct instrumental measurement of the lipid-free sample (δ^13^C_lipid-free_), but requires more time and effort. Additionally, in some (but not all^15,16^) cases, chemical lipid extraction can alter sample δ^15^N by removing amino acids^17^. Thus, to ensure comparable measurements of both δ^13^C and δ^15^N across tissues, it is recommended that two aliquots of each sample be analyzed: one non-lipid extracted (bulk) and the other with lipids removed^17^. The δ^15^N value of the non-lipid extracted aliquot (δ^15^N_bulk_) and the δ^13^C_lipid-free_ can then be interpreted together; however, this approach increases the time and effort required for sample preparation and doubles the analytical cost.

Lipid normalization is an alternative to chemical lipid extraction that allows δ^13^C_lipid-free_ to be approximated mathematically. This approach, sometimes called arithmetic or mathematical lipid correction, involves modeling the expected δ^13^C_lipid-free_ based on the lipid content of the bulk sample. These models have been widely used for multiple tissues from a variety of taxa and can be quite accurate in their estimation of δ^13^C_lipid-free_ (Supplementary Table 1). Typically, these models rely on the relationship between the carbon:nitrogen ratio of the non-lipid extracted sample (C:N_bulk_; a proxy for the lipid content of the sample^8,18^), and the difference between δ^13^C values of the lipid-free and non-lipid extracted aliquots of each sample (δ^13^C_lipid-free_ − δ^13^C_bulk_ = Δδ^13^C). This relationship tends to be species- and tissue-specific, thus, one limitation of lipid normalization models is that they must be parameterized for each species and tissue by performing chemical lipid extractions and comparing δ^13^C_bulk_ to δ^13^C_lipid-free_. Consequently, there are many species and tissues for which lipid normalization models are not available in the literature.

Tissue-specific isotope discrimination is another source of variability in stable isotope values that can impact diet reconstructions and the interpretation of δ^13^C and δ^15^N values. Stable isotope values in the tissues of an individual animal may vary substantially due to metabolic discrimination and differences in tissue composition^2,19^. Additionally, the degree of δ^13^C and δ^15^N discrimination between an animal’s diet and its tissues may be impacted by diet quality (i.e., protein and lipid content^20^). Finally, metabolic routing of different diet components (i.e., the pathways taken by dietary macromolecules such as proteins, fats, and carbohydrates during tissue synthesis and metabolism) impacts diet-tissue isotope discrimination and may mean that some tissues primarily reflect individual diet components (e.g., dietary protein), while others may be representative of whole diet^21^. Diet-tissue discrimination has been studied in a variety of animals and varies widely among species^22^. Thus, as with lipid normalization models, species- and tissue-specific discrimination factors are not currently available for many species.

Understanding diet-tissue discrimination, as well as the relationships between δ^13^C and δ^15^N of different tissues of individual animals, is critical for adequate interpretation of stable isotope studies. Furthermore, quantifying the degree of tissue-specific isotope discrimination allows researchers to more directly compare the results of studies analyzing δ^13^C and δ^15^N in different tissues. This is particularly valuable for comparing modern studies, which typically sample soft tissues, to studies of historic and paleoecology, for which bone collagen is the primary tissue available for analysis^23^. It is also important because analyses of various soft tissues provide different advantages. For many vertebrates, skin can be obtained using a biopsy, which is typically non-lethal and relatively non-invasive^24,25^. Muscle is often analyzed because this tissue has relatively slow metabolic turnover, can provide diet information over a period of weeks or months, and is the most commonly used vertebrate tissue in the published stable isotope literature^26,27^. Tissues with more rapid metabolic turnover rates can provide shorter-term information about diet, and are particularly informative when analyzed in tandem with tissues that turn over more slowly. Blood has a rapid turnover rate^26^, but obtaining it can be logistically difficult for some animals (e.g., free-ranging and subsistence harvested marine mammals). Liver also exhibits high metabolic turnover^26^, and can provide short-term diet information. Collection of liver samples can be logistically challenging, but may be feasible in instances where proper sampling and storage of blood are not.

Arctic marine mammals are ideal candidates for stable isotopic research. Biological systems in the Arctic have been historically understudied, and are currently undergoing rapid changes associated with warming of the regional climate^28,29^. Stable isotopes may provide a useful tool for understanding these changes. Examining δ^13^C and δ^15^N in marine mammal tissues can not only provide insight into the diet and movements of the animals themselves, but can also give important information about the food webs in which these animals feed^8^. Arctic marine mammals, including Pacific walruses (*Odobenus rosmarus divergens*), are important subsistence resources for Alaska Native communities, and monitoring the responses of these animals to climate change is critical to food security in the region. The overarching goal of this study was to address some of the factors that might confound the interpretation of stable isotope values in the tissues of Pacific walruses, with the goal of improving the ability of future research to draw conclusions about these animals and the food webs in which they feed. To accomplish this, δ^13^C and δ^15^N in walrus muscle, liver, skin, and bone collagen were analyzed to (1) assess the importance of lipid removal when comparing δ^13^C of various walrus tissues, (2) parameterize lipid normalization models for estimating Δδ^13^C based on C:N_bulk_, and (3) estimate tissue-specific discrimination factors for Pacific walruses.

## Methods

Skin, muscle, liver, and bone samples were collected from 30 adult walruses (20 males, 10 females) taken as part of the Alaska Native subsistence harvests on St. Lawrence Island, Alaska, from 2014–2016. Additional muscle (n = 95), liver (n = 5), and skin (n = 5) samples from the 2012–2016 subsistence harvests were analyzed to generate mathematical lipid-corrections and test the effects of chemical lipid extraction on δ^13^C and δ^15^N. Samples were collected in the field (typically in May in June) by Alaska Native subsistence hunters using guidelines developed by researchers at the Alaska Department of Fish and Game (ADF&G). At the time of harvest, skin, blubber and muscle were collected in a single, full-thickness sample, and a single, large section of the liver was taken. These tissues were frozen and shipped to ADF&G, in Fairbanks, Alaska, before being transferred to the University of Alaska Fairbanks, where they were stored at −80 °C. Bone samples were also collected by subsistence hunters at the time of harvest. Samples consisted mostly of cranium and mandible fragments; however, the skeletal element from which a sample was taken was not always identifiable. Samples that appeared to come from distal limb bones (i.e., carpals, tarsals, phalanges) were not used for this study, as the stable isotope values of these bones may not be representative of the skeleton as a whole^30^. Bones were cleaned of adherent tissue with a wire brush, dried, and stored at room temperature. All tissues used in this study were obtained under a letter of authorization to L. Horstmann from the United States Fish and Wildlife Service.

In preparation for stable isotope analysis, ~10 g of each soft tissue was subsampled, freeze-dried for 48 hours, and homogenized using a Wig-L-Bug^®^ grinding mill. Hair was typically relatively sparse on the skin samples, and surface hair was removed with a razor blade prior to homogenization. Any remaining pieces of hair were typically resistant to homogenization, thus remained intact and could be avoided during subsampling. Homogenized samples were divided into two equal parts. A 0.2–0.4 mg subsample was taken from one half and submitted for stable isotope analysis. The other half was lipid-extracted using methods modified from Folch *et al*.^14^ and Bligh and Dyer^13^. Briefly, samples were immersed in 2:1 chloroform:methanol and mixed thoroughly using a vortex mixer. Samples were then allowed to sit for 15 minutes before they were centrifuged and the chloroform:methanol was removed by pipet. This process was repeated until the supernatant remained clear, at which point samples were allowed to air dry overnight. A 0.2–0.4 mg subsample of the lipid-extracted sample was then submitted for stable isotope analysis.

Collagen was extracted from bone samples using the methods described by Misarti *et al*. (2009)^31^, as modified from Matheus (1995)^32^. A ~0.4 g subsample of solid, cortical bone was removed from each specimen using handheld cutting tools, cleaned in a sonic bath, and allowed to air dry for 1–2 days. Lipids were removed by soaking the bone in 2:1 chloroform:methanol for 8 hours and discarding the solvent. The mineral component of the bone was then removed using a mixture of 6N hydrochloric acid and ultrapure water. After demineralization, the sample was gelatinized at 65 °C, filtered through a 0.45 μm filter to remove any insoluble particles and non-collagen organic compounds, and freeze-dried. A 0.2–0.4 mg subsample of the resulting purified collagen was submitted for stable isotope analysis.

Stable carbon and nitrogen isotope values of tissue samples were analyzed in the Alaska Stable Isotope Facility at the University of Alaska Fairbanks, using a Costech ECS 4010 elemental analyzer and ThermoScientific Conflo IV, interfaced with a ThermoScientific DeltaV isotope ratio mass spectrometer. Stable isotopic compositions were calibrated relative to Vienna Pee Dee Belemnite (VPDB) and atmospheric nitrogen gas (air) using USGS40 and USGS41 as calibration standards. Results were reported in parts per thousand (‰) using δ notation. A commercially available peptone standard (No. P-7750 bovine based protein, Sigma Chemical Company, lot #76f-0300; δ^13^C: −15.8‰, δ^15^N: 7.0‰, %C: 44.3, %N: 15.3) was analyzed as a check standard after every 10 samples to estimate uncertainty. Precision (±1 standard deviation; SD) of these analyses was ±0.1‰ for both δ^13^C and δ^15^N, based on repeated measurements of this standard across all analytical runs (n = 81). Measurements were accurate (±1 SD) to within ±0.03‰ for δ^13^C and ±0.05‰ for δ^15^N, based on differences between observed and known values of the peptone standard. Sample composition (%C and %N) was measured during stable isotope analysis, and was used to calculate the C:N ratio of the sample using the formula:

$$C:N=( \% C)/( \% N)$$

Paired t-tests were used to determine whether changes in δ^13^C and δ^15^N associated with lipid-extraction (Δδ^13^C) were significant for walrus muscle (n = 125), liver (n = 35), and skin samples (n = 35). To generate arithmetic lipid-corrections, Δδ^13^C was regressed against the carbon:nitrogen ratio of the non-lipid extracted sample (C:N_bulk_). Lipid-correction models from the literature were fitted to the relationship, using the nlstools package in R^33^ to estimate parameters. These non-linear least squares models assume that residual error is normally distributed, and this assumption was met for all three tissue types.

The first model fitted was Equation 1a from Logan *et al*. (2008)^34^, which was derived from McConnaughey and McRoy’s (1979)^8^ Equation 1:

1 $${\Delta {\rm{\delta }}}^{13}{\rm{C}}=\frac{a\,\ast \,C:{N}_{bulk}+b}{C:{N}_{bulk}+c}$$

In this model, the x-intercept (−*b/a*) represents the estimated C:N_lipid-free_, whereas the maximum Δδ^13^C is represented by *a*. The second model tested was Equation 2 from Logan *et al*. (2008)^34^, as derived from Fry (2002)^35^:

2 $${\Delta {\rm{\delta }}}^{13}{\rm{C}}=p-\frac{p\,\ast \,f}{C:{N}_{bulk}}$$

The model-estimated C:N_lipid-free_ in this equation is represented by ƒ, while *p* represents protein-lipid δ^13^C discrimination. The third model tested was Equation 3 from Logan *et al*. (2008)^34^:

3 $${\Delta {\rm{\delta }}}^{13}{\rm{C}}={\beta }_{0}+{\beta }_{1}\,\ast \,\mathrm{ln}(C:{N}_{Bulk})$$

In this model, the estimated C:N_Lipid-free_ is represented by ${e}^{\frac{-{\beta }_{0}}{{\beta }_{1}}}$. Finally, a linear model was fitted to the data for comparison with the above equations.

4 $${\Delta {\rm{\delta }}}^{13}{\rm{C}}={\rm{a}}+{\rm{b}}\,\ast \,C:{N}_{bulk}$$

Predictive error was assessed using leave-one-out cross validation^36^. For each model, this technique was used to calculate mean squared error (MSE), mean absolute error (MAE), and the proportion of the samples for which the arithmetically estimated Δδ^13^C was within 0.5‰ of the measured Δδ^13^C. This 0.5‰ value represents roughly twice the measurement error commonly reported in ecological studies measuring δ^13^C, thus differences below this threshold are often not considered biologically important^37,38^. These metrics were compared to assess model fit, with the best fitting model defined as that with the lowest MSE and MAE, and the greatest proportion of samples for which Δδ^13^C was estimated to within 0.5‰ of measured Δδ^13^C.

Comparisons among walrus tissues were made using δ^13^C_lipid-free_. Because chemical lipid-extraction may impact stable nitrogen isotope values^34^, δ^15^N values from non-lipid extracted samples were used for these analyses. Linear mixed effects models were used to test whether δ^13^C_lipid-free_ and δ^15^N differed among muscle, liver, skin, and bone collagen of 30 individual walruses. These models assume a Gaussian error distribution, and this assumption was met for all tissues. For each model, tissue was entered as a fixed effect. Individual was included as a by-subject random intercept to account for relationships among the isotope values of tissues from individual walruses. Models were fitted using the R package lme4^39^, and p-values were obtained by likelihood ratio tests of the full model with the effect of tissue against the null model, which did not include tissue as an effect. Significance was assessed using an alpha of 0.05, and residuals were visually inspected for deviations from normality and homoscedasticity. Tukey’s Honest Significant Difference *post hoc* tests with a Holm-Bonferroni correction^40^ were conducted in the multcomp R package^41^ to examine differences among tissues. Tissue samples from female walruses were only available in 2016, and only 10 of the 30 animals sampled for this study were female. Additionally, there is no evidence to support the assumption that differences in stable isotope values between males and females observed in this study would remain consistent seasonally, inter-annually, and across broader time scales, thus sex of sampled walruses was not included in the models presented in here. However, visual examination of the data revealed apparent differences between the tissue stable isotope values of male and female walruses included in this study, thus additional linear mixed effects models including sex were run. The results of these models are presented and interpreted in the online supplementary material (Supplementary Discussion 1). All statistical analyses were conducted using R version 3.4.1^42^ with RStudio version 1.0.153^43^.

## Results

Chemical lipid extraction changed the C:N ratios, δ^13^C, and/or δ^15^N of walrus tissues (Table 1). Paired t-tests indicated that chemical lipid extraction significantly increased the δ^13^C values of walrus liver (mean change ± 1 SD = 0.9 ± 0.3‰, t_34_ = 17.42, p < 0.001) and skin (mean change ± 1 SD = 1.9 ± 1.8‰, t_34_ = 6.18, p < 0.001), but walrus muscle δ^13^C was unchanged (mean change ± 1 SD = 0.0 ± 0.5‰, t_124_ = 1.07, p = 0.287). Lipid extraction significantly increased the δ^15^N of muscle (mean change ± 1 SD = 0.1 ± 0.3‰, t_124_ = 3.05, p = 0.002) and liver (mean change ± 1 SD = 0.2 ± 0.2‰, t_34_ = 6.50, p < 0.001), whereas changes to skin δ^15^N (Δδ^15^N) were not statistically significant (mean change ± 1 SD = 0.1 ± 0.3‰, t_34_ = 1.46, p = 0.155). Regardless of whether mean δ^15^N was impacted by lipid extraction, the difference between δ^15^N_bulk_ and δ^15^N_lipid-free_ (Δδ^15^N = δ^15^N_bulk_ − δ^15^N_lipid-free_) of a sample from an individual animal was frequently well beyond the range of analytical precision (muscle Δδ^15^N range = −0.7–1.0‰; liver Δδ^15^N range = −0.1–0.5‰; skin Δδ^15^N range = −0.6–0.8‰).

**Table 1):** Mean (±1 SD) δ^13^C and δ^15^N, as well as C:N ratios, for non-lipid extracted and lipid extracted walrus muscle, liver, and skin (n = 30). Bone collagen is a purified protein matrix, so this tissue was not lipid extracted.

		δ^13^C_bulk_ (‰)	δ^15^N_bulk_ (‰)	C:N_bulk_	δ^13^C_lipid-free_ (‰)	δ^15^N_lipid-free_ (‰)	C:N_lipid-free_
Muscle	*Mean* (±*1 SD)*	−17.0 ± 0.4	12.6 ± 0.5	3.5 ± 0.2	−16.8 ± 0.2	12.6 ± 0.5	3.3 ± 0.2
	*Median*	−16.9	12.6	3.4	−16.8	12.5	3.3
	*Range*	−17.9–16.4	11.6–13.6	3.2–4.0	−17.5–−16.2	11.6–13.6	3.1–3.8
Liver	*Mean* (±*1 SD)*	−18.3 ± 0.5	12.6 ± 0.8	4.7 ± 0.5	−17.3 ± 0.4	12.8 ± 0.8	4.0 ± 0.4
	*Median*	−18.1	12.4	4.6	−17.3	12.7	3.9
	*Range*	−19.4–−17.7	11.6–14.1	3.9–6.0	−18.3–−16.6	11.8–14.1	3.4–4.6
Skin	*Mean* (±*1 SD)*	−16.7 ± 2.1	14.7 ± 0.6	4.1 ± 1.9	−14.8 ± 0.5	14.8 ± 0.6	3.1 ± 0.2
	*Median*	−16.2	14.7	3.3	−14.7	14.8	3.1
	*Range*	−21.3	13.6–15.7	2.7–10.5	−15.8–−13.9	13.6–16.0	2.8–3.4
Bone Collagen	*Mean* (±*1 SD)*	−14.8 ± 0.3	12.2 ± 0.7	2.9 ± 0.1	—	—	—
	*Median*	−14.7	12.2	2.9	—	—	—
	*Range*	−15.6–−14.2	10.7–13.8	2.7–3.1	—	—	—

**Figure 1:**
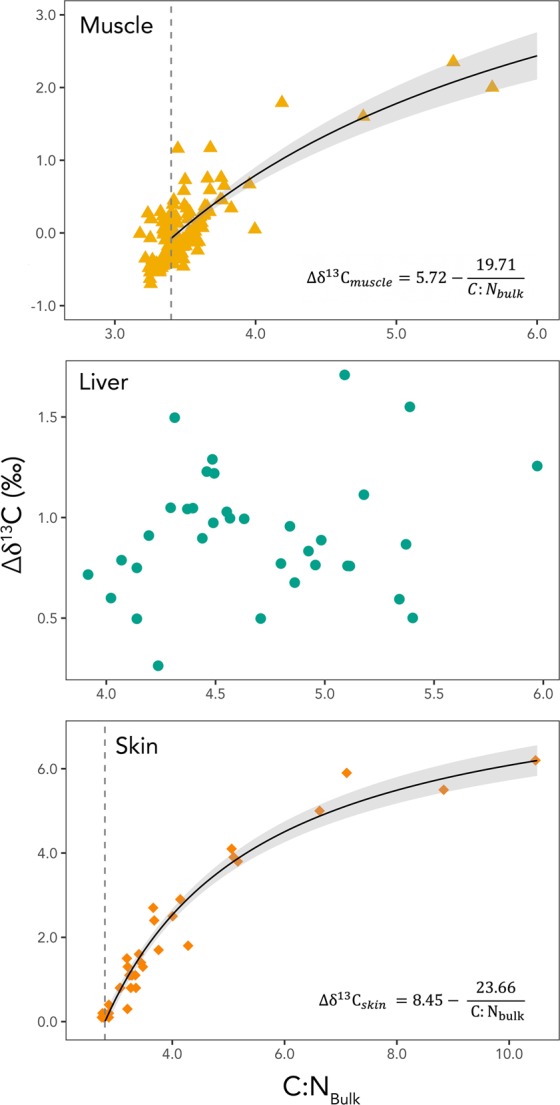
Non-lipid extracted carbon:nitrogen ratios (C:N_bulk_) of walrus muscle (top, yellow triangles, n = 125), liver (middle, green circles, n = 35), and skin (bottom, orange diamonds, n = 35) plotted against the change in δ^13^C due to chemical lipid extraction (Δδ^13^C). Equations reflect the best fitting lipid-normalization models for muscle and skin, and solid black lines are predicted values for these models. Gray shaded areas represent 95% confidence envelopes for the predicted lines. Vertical dashed lines represent the carbon:nitrogen ratio of the lipid-free tissue (C:N_lipid-free_) as predicted by the best-fitting lipid normalization model. It is important to note that the axes of each plot are scaled differently.

Lipid-normalization models were parameterized for walrus muscle, liver, and skin, but not for bone collagen, which is a purified protein. Of the three tissues examined in this portion of the study, only muscle and skin showed a relationship between Δδ^13^C and C:N_bulk_ (Table 2, Fig. 1). Liver Δδ^13^C was not related to C:N_bulk_, thus a mathematical lipid correction could not be generated. For muscle, evaluation of model fit indicated that Equations 1–3 performed almost identically in their predictions of Δδ^13^C (Table 2, Fig. 1). Because lipid normalization models are primarily useful for predicting Δδ^13^C of samples with high C:N_bulk_ values, the fit of the three models was reevaluated by performing leave-one-out cross validation using only samples with C:N_bulk_ > 3.75 (n = 10). When model fit was considered for only these 10 samples, Equation 2 best predicted Δδ^13^C (MSE = 0.167, MAE = 0.307, P_0.5_ = 0.80), followed by Equations 3 (MSE = 0.192, MAE = 0.318, P_0.5_ = 0.70) and 1 (MSE = 0.198, MAE = 0.338, P_0.5_ = 0.70). The parameters *p* and ƒ were estimated for Equation 2 using walrus muscle δ^13^C values from this study, resulting in the following equation:

5 $${\Delta {\rm{\delta }}}^{13}{{\rm{C}}}_{muscle}=5.72-\frac{5.72\,\ast \,3.45}{C:{N}_{bulk}}$$

**Table 2:** Lipid normalization models, equations, and parameters derived from walrus muscle samples (n = 125), as well as estimated C:N_lipid-free_ for each model. Mean squared error (MSE), mean absolute error (MAE), and the percentage of samples predicted to fall within 0.5‰ of lipid extracted values (Pred_0.5_) were calculated from leave-one-out cross validation and used to assess model fit. Models are ordered from best (top) to worst (bottom) fit (best fit = lowest MSE, lowest MAE, highest Pred_0.5_).

Model	Equation	Parameters (95% CI)	Estimated C:N_lipid-free_	MSE	MAE	Pred_0.5_ (%)
Logan *et al*. (2008)^34^ Eqn 2	${\Delta {\rm{\delta }}}^{13}{\rm{C}}=p-\frac{p\,\ast \,f}{C:{N}_{Bulk}}$	*p* = 5.72 (4.96; 6.48)	*f* = 3.4	0.093	0.242	91.2
		*f* = 3.45 (3.41; 3.48)				
Logan *et al*. (2008)^34^ Eqn 3	${\Delta {\rm{\delta }}}^{13}{\rm{C}}={\beta }_{0}+{\beta }_{1}\ast \,\mathrm{ln}(C:{N}_{Bulk})$	*β*_0_ =−6.12 (−6.95; −5.28)	${e}^{\frac{-{\beta }_{0}}{{\beta }_{1}}}=3.5$	0.095	0.243	92.0
		*β*_1_ = 4.94 (4.27; 5.61)				
Logan *et al*. (2008)^34^ Eqn 1a	${\Delta {\rm{\delta }}}^{13}{\rm{C}}=\frac{a\ast C:{N}_{Bulk}+b}{C:{N}_{Bulk}+c}$	*a* = 6.34 (1.71; 10.96)	$\frac{-b}{a}=3.4$	0.096	0.245	91.2
		*b* =−21.84 (−37.83; −5.85)				
		*c* = 0.48 (−3.11; 4.07)				
Linear	${\Delta {\rm{\delta }}}^{13}{\rm{C}}=a+b\ast (C:{N}_{Bulk})$	*a* = 1.19 (1.02; 1.35)	${x}_{int.}=3.5$	0.102	0.250	92.0
		*b* = −4.20 (−4.78; −3.61)

Which was simplified to:

5a $${\Delta {\rm{\delta }}}^{13}{{\rm{C}}}_{muscle}=5.72-\frac{19.71}{C:{N}_{bulk}}$$

The mean and median differences between the observed Δδ^13^C and the values predicted by this model were both 0.2‰. The maximum difference between the observed and predicted Δδ^13^C for a given individual was 1.2‰.

For walrus skin, the relationship between Δδ^13^C and C:N_bulk_ was best explained by Equation 2, which predicted Δδ^13^C to within 0.5‰ for >85% of samples (Table 3, Fig. 1). Walrus skin δ^13^C values from this study were used to estimate the parameters *p* and ƒ, which generated the following equation:

6 $${\Delta {\rm{\delta }}}^{13}{{\rm{C}}}_{skin}=8.45-\frac{8.45\,\ast \,2.80}{\,C:{{\rm{N}}}_{{\rm{bulk}}}}$$

**Table 3:** Lipid normalization models, equations, and parameters with 95**%** confidence intervals (95**%** CI) derived from walrus skin samples (n = 35), as well as estimated C:N_lipid-free_ for each model. Mean squared error (MSE), mean absolute error (MAE), and the percentage of samples predicted to fall within 0.5**‰** of lipid extracted values (Pred_0.5_) were calculated from leave-one-out cross validation and used to assess model fit. Models are ordered from best (top) to worst (bottom) fit (best fit = lowest MSE, lowest MAE, highest Pred_0.5_).

Model	Equation	Parameters (95% CI)	Estimated C:N_lipid-free_	MSE	MAE	Pred_0.5_ (%)
Logan *et al*. (2008)^34^ Eqn 2	${\Delta {\rm{\delta }}}^{13}{\rm{C}}=p-\frac{p\,\ast \,f}{C:{N}_{Bulk}}$	*p* = 8.45 (7.92; 8.98)	*f* = 2.8	0.157	0.295	85.7
		*f* = 2.80 (2.74; 2.87)				
Logan *et al*. (2008)^34^ Eqn 1a	${\Delta {\rm{\delta }}}^{13}{\rm{C}}=\frac{a\,\ast \,C:{N}_{Bulk}+b}{C:{N}_{Bulk}+c}$	*a* = 9.33 (7.63; 11.04)	$\frac{-b}{a}=2.8$	0.161	0.298	80.0
		*b* =−25.81 (−30.17; −21.45)				
		*c* = 0.73 (−0.63; 2.09)				
Logan *et al*. (2008)^34^ Eqn 3	${\Delta {\rm{\delta }}}^{13}{\rm{C}}={\beta }_{0}+{\beta }_{1}\,\ast \,\mathrm{ln}(C:{N}_{Bulk})$	*β*_0_ = −4.94 (−5.59; −4.29)	${e}^{\frac{-{\beta }_{0}}{{\beta }_{1}}}=2.6$	0.249	0.376	68.6
		*β*_1_ = 5.13 (4.66; 5.61)				
Linear	${\Delta {\rm{\delta }}}^{13}{\rm{C}}=a+b\,\ast \,(C:{N}_{Bulk})$	*a* = 0.93 (0.78; 1.06)	${x}_{int.}=2.0$	0.679	0.62	45.7
		*b* =−1.87 (−2.46; −1.25)				

Which was simplified to:

6a $${\Delta {\rm{\delta }}}^{13}{{\rm{C}}}_{skin}=8.45-\frac{23.66}{\,C:{{\rm{N}}}_{{\rm{bulk}}}}$$

For this model, the mean and median differences between the observed and predicted Δδ^13^C values were 0.3‰ and 0.2‰, respectively. The maximum difference between the observed and predicted Δδ^13^C for a given individual was 1.1‰.

**Figure 2:**
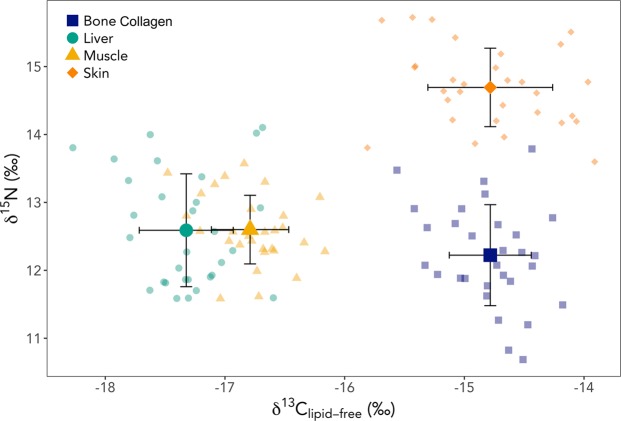
Scatterplot of δ^13^C_lipid-free_ and δ^15^N values for muscle (yellow triangles), liver (green circles), skin (orange diamonds), and bone collagen (blue squares) from 30 individual walruses. Each walrus is represented by a complete set of tissues (muscle, liver, skin, and bone collagen). Mean values are represented by larger, darker symbols. Error bars represent ±1 SD from the mean.

Linear mixed effects models indicated that δ^13^C_lipid-free_ and δ^15^N differed significantly among walrus tissues (δ^13^C_lipid-free_: χ^2^_3_ = 282.87, p < 0.001; δ^15^N: χ^2^_3_ = 193.86, p < 0.001; Tables 1 and 4, Fig. 2). Liver had the lowest δ^13^C_lipid-free_ values (Tables 1, 4, and 5, Fig. 2). The output from the linear mixed effects models indicated that muscle δ^13^C_lipid-free_ was, on average, 0.5 (95% CI: 0.3–0.7)‰ higher than liver. Skin and bone collagen were enriched in ^13^C by an additional 2.0 (95% CI: 1.8–2.2)‰ compared with muscle. *Post hoc* tests showed that the δ^13^C_lipid-free_ of all tissues differed significantly, except for skin and bone collagen (Table 5). Bone collagen had the lowest δ^15^N values (Tables 1, 4, and 5, Fig. 2). Liver and muscle were enriched in ^15^N by 0.4 (95% CI: 0.2–0.6)‰ in relation to bone collagen. Skin δ^15^N values were an average of 2.1 (95% CI: 1.9–2.3)‰ higher than those of liver and muscle. For δ^15^N, all tissues were significantly different, except for muscle and liver (Table 5).

**Table 4:** Coefficient estimates and standard errors for the linear mixed effects models testing the relationship between walrus tissue type (muscle, liver, skin, and bone) and δ^13^C_lipid-free_ and δ^15^N. Both models included a random intercept for individual, to account for relationship among stable isotope values for tissues from individual walruses.

	δ^13^C_lipid-free_ (‰)		δ^15^N (‰)	
	Estimate	Standard Error	Estimate	Standard Error
Muscle	−16.8	0.1	12.6	0.1
Liver	−17.3	0.1	12.6	0.1
Skin	−14.8	0.1	14.7	0.1
Bone	−14.8	0.1	12.2	0.1

**Table 5:** Mean differences and 95% confidence intervals (95% CI) in tissue δ^13^C_lipid-free_ (top right) and δ^15^N (bottom left), as estimated by the linear mixed effects models and *post hoc* tests. Asterisks indicate significant differences (p < 0.05).

	δ^13^C_lipid-free_ (‰): Mean difference (95% CI)			
	Bone	Liver	Muscle	Skin
Bone	—	2.5 (2.3–2.7)*	2.0 (1.8–2.2)*	0.0 (–0.2–0.2)
Liver	0.4 (0.2–0.6)*	—	0.5 (0.3–0.7)*	2.5 (2.3–2.7)*
Muscle	0.4 (0.2–0.6)*	0.0 (–0.2–0.2)	—	2.0 (1.8–2.2)*
Skin	2.5 (2.3–2.7)*	2.1 (1.9–2.3)*	2.1 (1.9–2.3)*	—
	**δ**^**15**^**N (‰): Mean difference (95% CI)**			

## Discussion

Accounting for sample lipid content (either by mathematical lipid correction or lipid extraction) and understanding tissue-specific discrimination are important for properly interpreting the results of stable isotope analyses of animal tissues. This is particularly true when δ^13^C and δ^15^N values are used to make quantitative diet reconstructions^44,45^, as well as when making decisions about which tissue to analyze or comparing stable isotope values among different tissues^46^. The results of this study provide quantitative estimates of the effects of chemical lipid extraction on δ^13^C and δ^15^N in walrus muscle, liver, and skin, lipid normalization models for walrus muscle and skin, and tissue-specific stable isotope discrimination factors for walrus muscle, liver, skin, and bone collagen.

### Effects of Lipids on δ^13^C

Many studies have demonstrated the negative relationship between δ^13^C and lipid content in animal tissues^1,17,34,44^. In this study, chemical lipid extractions significantly increased the δ^13^C values of walrus liver and skin, indicating that lipids affected δ^13^C in these tissues. Muscle δ^13^C remained unchanged, likely because marine mammal muscle is typically comprised of lean protein with little lipid content^47–49^. Lipids are stored primarily in the blubber layer in these animals, rather than interspersed among the muscles. Depending on the degree of precision desired, future studies measuring δ^13^C in walrus muscle may choose to forego lipid extraction and/or lipid normalization altogether. Because an arithmetic lipid correction could not be generated for walrus liver, lipids should be chemically extracted from this tissue if liver δ^13^C is to be used for future studies.

Mathematical lipid correction requires that the change in δ^13^C associated with lipid removal (Δδ^13^C) be statistically related to the carbon:nitrogen ratio of the bulk sample. For walrus skin, Δδ^13^C was proportional to C:N_bulk_, such that higher C:N ratios prior to lipid extraction were associated with larger changes to δ^13^C values as a result of extraction. Though chemical lipid extraction did not affect the mean δ^13^C of walrus muscle, samples with high C:N_bulk_ were associated with the largest changes in δ^13^C. C:N_bulk_ is indicative of the lipid content^8,18^, thus, as expected, samples with a greater lipid content exhibited a larger change in δ^13^C after the lipids were removed. The top-performing model for walrus muscle (Equation 5a) was parameterized from Equation 2 in Logan *et al*. (2008)^34^, using experimental data. In this equation, the C:N_lipid-free_ can be estimated by the term *f*, which resulted in a value of 3.45 (95% confidence interval [CI]: 3.41–3.48), indicating that a muscle sample with a C:N_bulk_ of ≤3.45 is comprised entirely of lean protein and need not be lipid extracted or corrected. Walrus muscle analyzed in this study appears to have contained very few lipids, with the model output indicating that more than 40% of samples had a carbon:nitrogen ratio less than or equal to 3.45. Some samples did contain a higher proportion of lipids, possibly as a result of the location from which the sample was taken (e.g., close to the blubber layer, where lipids may have leached into the muscle). Future studies may wish to sample muscle from locations far from the blubber to reduce the amount of lipids in the tissue. In instances where this is not possible, the equation presented here may be used to estimate Δδ^13^C of muscle with a greater-than-average lipid content (C:N_bulk_ > 3.45).

The C:N ratio of walrus skin (a proxy for lipid content^8,18^) ranged widely (C:N_bulk_ range = 2.7–10.5), and Δδ^13^C was proportional to C:N_bulk_, making this tissue particularly well-suited for mathematical lipid correction. The top-performing lipid normalization model (Equation 6a) was adapted from the Logan *et al*. (2008)^34^ derivation of the Fry (2002)^35^ mass balance equation (Equation 2), which uses the C:N_bulk_, C:N_lipid-free_, and a term describing protein-lipid δ^13^C discrimination to estimate Δδ^13^C. The latter two parameters were estimated from the experimental data, providing valuable information about the characteristics of walrus skin. The C:N_lipid-free_ parameter from the fitted model had a value of 2.80 (95% CI: 2.74–2.87), indicating that a skin sample with a C:N_bulk_ of ~2.8 is already composed of lean protein and need not be lipid-extracted or corrected. The lipid-protein discrimination parameter was estimated to be 8.45, which was greater than published estimates for various fishes and invertebrates^34^, but similar to estimates for baleen whale skin and blubber^38^. The best-fitting model was effective at predicting Δδ^13^C for walrus skin samples based on a wide range of C:N_bulk_ values. These predicted values were within 0.5‰ for greater than 85% of samples. This level of precision is likely acceptable for many ecological studies; however, in situations where researchers seek to address questions requiring greater precision, chemical lipid extraction may be necessary.

Removal of lipids from walrus tissues sometimes resulted in unexpected changes in δ^13^C. For some samples, δ^13^C_lipid-free_ was lower than δ^13^C_bulk_, resulting in a negative value for Δδ^13^C. Chemical lipid extraction should not reduce δ^13^C, therefore these negative Δδ^13^C values likely resulted due to analytical error, within-tissue heterogeneity in δ^13^C, and/or incomplete homogenization of tissue samples before stable isotope analysis. Because only a small subsample (~0.2 mg) is analyzed for stable isotopes, it is possible or even likely that two subsamples taken from an incompletely homogenized tissue may differ in their composition (i.e., fatty acid and amino acid compositions), resulting in δ^13^C and δ^15^N differences. Most of the negative Δδ^13^C values were small (~−0.1‰); however, some muscle samples had δ^13^C_lipid-free_ values that were much lower (~−0.6‰) than their δ^13^C_bulk_ values. Ensuring samples are completely homogenized before subsampling for isotope analysis may help reduce this type of error in future studies. Complete homogenization is particularly important because differences in stable isotope values may exist at small spatial scales within a tissue. Todd *et al*. (2010) found little variability in pinnipeds skin and muscle sampled at different sites on the body^50^; however, Wild *et al*. (2018) described differences in both δ^13^C and δ^15^N among cetacean skin layers^51^, indicating that this tissue represents a dietary time series, and stable isotope values may not be homogenous at different depths within a sample. Improper subsampling or incomplete homogenization prior to analysis could thus result in δ^13^C and δ^15^N differences driven by these spatial variations in isotope values within the skin. Further investigations into how δ^13^C and δ^15^N vary within individual tissues are warranted. In particular, it would be valuable to examine variations among different muscle groups, as well as differences between glycolytic and oxidative muscles.

### Effects of Chemical Lipid Extraction on δ^15^N

A substantial drawback to chemical lipid extractions is that they may alter δ^15^N values, thus, a lipid extracted and non-lipid extracted aliquot of each sample must be run to ensure comparability across tissues and studies of both δ^13^C and δ^15^N values. Sotiropoulos *et al*. (2004)^17^ hypothesized that δ^15^N increases as the organic solvent removes amino acids contained within structural lipids; however, as Ryan *et al*. (2012)^38^ also noted for cetacean skin and blubber samples, changes in δ^15^N observed in our study were not unidirectional. The δ^15^N values of some walrus skin samples decreased by as much as 0.6‰, while others increased by as much as 0.8‰, indicating that the removal of ^15^N was not uniform across samples. The same pattern was observed for walrus muscle and liver, though the observed changes in δ^15^N were smaller. This may have resulted from the removal of amino acids from the sample during chemical lipid extraction^17^, but may also have been related to incomplete homogenization during sample processing and/or variation in sample lipid content. The effects of lipid extractions on δ^15^N in this study suggest that, for studies using chemical lipid extraction on walrus tissues, isotope values of samples must be analyzed both before and after extraction. This makes chemical lipid extraction at least twice as expensive as mathematical lipid correction. Though lipid extraction with chloroform:methanol did not significantly alter the mean δ^15^N values of walrus skin samples used in this study, the δ^15^N of the lipid-free samples was often different enough from the bulk sample (up to 0.8‰) to alter interpretation of the results. Such changes to δ^15^N would be particularly problematic for studies using mixing models to estimate animal diet^52,53^. In the future, researchers should assess the level of precision required to investigate their hypotheses, as well as the number of samples they are able to run, if laboratory costs are a limiting factor, before deciding whether to use chemical lipid extractions or the arithmetic lipid-correction for walrus tissues.

### Tissue-specific δ^13^C and δ^15^N Discrimination

Differences in δ^13^C and δ^15^N among animal tissues arise from multiple factors that impact stable isotope values, including tissue-specific isotope discrimination^2,46,54^, inter-tissue variability in turnover rates^20,27,46^, and differences in metabolic routing of dietary macromolecules, such as proteins, fats, and carbohydrates^20,21,55^. Disentangling the effects of these different processes is difficult, and many controlled feeding experiments have investigated the role that each of these processes plays in determining the stable isotope values of individual tissues within an organism^27,46,54,56^. For studies of free-ranging animals, these processes must be considered together and the likely importance of each weighed when interpreting stable isotope data. Though the specific factors driving inter-tissue variability in δ^13^C and δ^15^N may remain unknown, general patterns in the differences among tissues can be observed and compared with those reported in the literature. For example, the −0.5 (95% CI: −0.7–−0.3)‰ offset in δ^13^C_lipid-free_ between walrus muscle and liver in the present study appears to be standard among marine mammals, for which reported values range from −0.8 to −0.1‰ (Supplementary Table 2 and references therein). Similarly, the +2.0 (95% CI: 1.8–2.2) ‰ difference between muscle and bone collagen δ^13^C_lipid-free_ is typical of marine mammals (range: 1.3–2.8‰; Supplementary Table 2 and references therein). In contrast, walrus skin exhibits unusually high enrichment in ^13^C and ^15^N (+2.0 [95% CI: 1.8–2.2] ‰ and +2.1 [95% CI: 1.9–2.3]‰, respectively) as compared with muscle values (published ranges: δ^13^C_lipid-free_ = −0.8–1.5‰; δ^15^N = −0.1–1.2‰; Supplementary Table 2 and references therein). Yurkowski *et al*. (2015)^57^ measured δ^13^C and δ^15^N in skin (n = 20) and liver (n = 19) of Atlantic walruses (*Odobenus rosmarus rosmarus*). Liver δ^13^C_lipid-free_ averaged 0.4‰ lower than muscle δ^13^C_lipid-free_, closely matching the average difference of −0.5 (95% CI: −0.7–−0.3)‰ observed in this study. In contrast, Atlantic walrus liver δ^15^N was 0.4‰ greater than muscle δ^15^N, as compared to a difference of 0.0 (95% CI: −0.2–0.2)‰ in our study. The contrasting results for δ^15^N may have arisen due to a variety of factors, including sample size, timing of sample collection, and differences in the physiology, ecology, diet, and seasonal movements of Pacific and Atlantic walruses.

**Figure 3:**
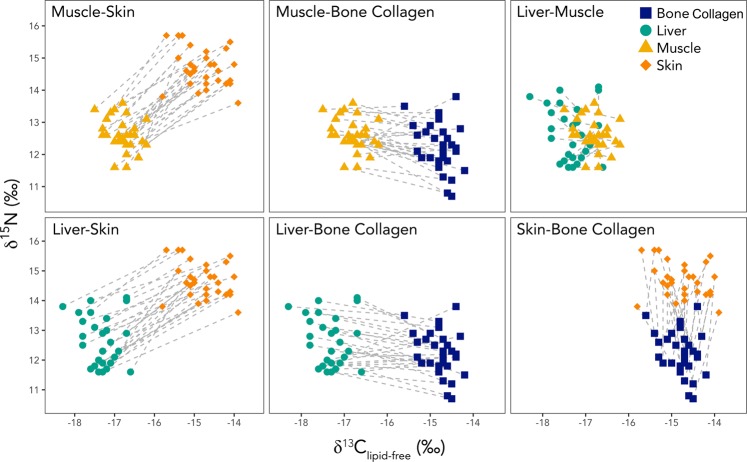
Comparisons of δ^13^C_lipid-free_ and δ^15^N for muscle (yellow triangles), liver (green circles), skin (orange diamonds), and bone collagen (blue squares) from 30 individual walruses. Each walrus is represented by a complete set of tissues (muscle, liver, skin, and bone collagen). Paired data points from individual walruses are connected by dashed lines. It is important to note that the axes of each plot on identical scales.

Examining the differences between paired samples from individual walruses on a tissue-by-tissue basis can provide insight into the factors driving inter-tissue variability in δ^13^C_lipid-free_ and δ^15^N (Fig. 3). In our study, individual walruses with higher δ^13^C_lipid-free_ and δ^15^N in one tissue generally had high δ^13^C_lipid-free_ and δ^15^N in their other tissues. When lines are drawn between the paired tissue samples (Fig. 3), the lines are mostly parallel and generally of the same length. This suggests that the differences between these tissues are likely driven primarily by tissue-specific discrimination or metabolic routing, which would be expected to produce a consistent offset between the stable isotope values of individual tissues. Comparisons involving liver are the exception to this general pattern, with more variability in the differences between tissues from individual animals (Fig. 3); this is most apparent in the comparison between liver and muscle. Liver exhibits a small, but generally consistent offset from muscle towards more negative δ^13^C_lipid-free_; however, the differences between liver and muscle are highly variable in their magnitude and direction. The same is true, to a lesser extent, for the liver-skin and liver-bone collagen comparisons. This variability in the offset between paired tissue samples likely reflects differences in turnover rate among tissues. Comparisons between tissues with rapid turnover rates and those with longer turnover rates are more likely to result in isotopic variability among tissues due to the contrast between the short-term diet signal represented by the tissue with rapid turnover, and the integrated, longer-term signal reflected in the tissue with slow turnover. When the difference in turnover rates is large, and the relative importance of tissue-specific discrimination and metabolic routing are smaller, turnover rate will likely play a greater role in determining the magnitude and direction of differences between tissue samples from individual animals.

Variation in turnover rate alone will not drive differences in the δ^13^C and δ^15^N of animal tissues. In the absence of other sources of variability in stable isotope values (i.e., tissue-specific discrimination and metabolic routing), animals consuming a monotonous diet would have consistent stable isotope values throughout their bodies, regardless of turnover rate. An animal’s diet must change for tissue turnover rates to drive differences in δ^13^C and δ^15^N among tissues. For walruses in this study, differences in δ^13^C_lipid-free_ and δ^15^N of liver and muscle likely resulted from changes in diet (or prey isotope values) associated with the annual migration. Pacific walrus migration is sex-segregated^58^. The entire population winters together in the Bering Sea. In the summer, females and juveniles move northward into the Chukchi Sea, whereas most males move in the coastal waters of the Bering Sea, including Bristol Bay and the Russian coastline. Female walruses had liver δ^15^N values that were 1.4 (95% CI: 1.0–1.8) ‰ greater than those of males; whereas female δ^15^N was only 0.7 (95% CI: 0.3–1.1) to 0.8(95% CI: 0.4–1.2)‰ greater than that of males for all other tissues (Supplementary Discussion 1). Stomach content analysis indicates that the diets of male and female walruses are essentially identical when they are in the same location^59^, thus these sex-related differences are probably driven by changes in diet during the summers, when males and females are separated. These differences likely result from shifts in diet composition and geographic variability in baseline δ^15^N values, and provide further support for the hypothesis that the observed differences in isotope values between liver and other tissues were driven primarily by differences in turnover rate.

The relative turnover rates of animal tissues have been the subject of many studies^60^. Though direct estimates are not available for walruses, the turnover rates of muscle, liver, skin, and bone collagen have been established for many other species and typically follow the order liver <muscle/skin <bone collagen^27,46^. Turnover rate is fastest in liver, with a typical reported half-life of <10 days^26,27,46,61^, whereas bone collagen turns over more slowly and can represent an average of years of foraging^26,27,62,63^. The turnover rate of muscle is intermediate, with a half-life that is on the order of weeks to months^26,27,61^. When considering values from the published literature, it is critical to note that the rate of tissue turnover is directly related to the animal’s mass-specific metabolic rate^64^, and is proportional to body size^60^. Most of the studies cited above measured tissue turnover in small animals (e.g., rodents, small birds) that are readily studied in the lab. These animals have high mass-specific metabolic rates, and their tissues almost certainly turn over much faster than those of larger animals. For example, the stable carbon isotope half-lives of liver (37.3 days) and muscle (178.7 days) in alpacas (*Lama pacos*) are approximately six times those reported (6.4 days and 27.6 days, respectively) for gerbils^46,64^. Domestic cattle (*Bos taurus*) have similarly slow δ^13^C and δ^15^N turnover in their skeletal muscle (~134–157 days^65^). Tissue turnover rates in walruses are likely to be much closer to those of larger mammals than the values estimated for small animals, and may be even slower given their large body size.

In contrast to liver, muscle, and bone collagen, the metabolic turnover of skin is not well studied. Most estimates in the literature come from studies of marine mammals, for which skin biopsy is a commonly used sampling method^66^. Estimates for cetacean skin turnover range from roughly one to six months^67–70^. Research by Wild *et al*. (2018)^51^ showed that cetacean skin, which grows continuously at its innermost margin and is sloughed off upon reaching the surface, contains a time series of stable isotope values that may reflect diet over a longer period of time. They suggested that the inner layer of the skin alone might represent diet from an entire summer feeding period (~4 months). It is important to note that the structure and function of cetacean skin is different from that of pinnipeds^58,71–73^, and these estimates might not be appropriate for walruses.

Tissue-specific stable isotope discrimination typically results from differences in the chemical composition of tissues. Proteins that differ in the relative abundance of their constituent amino acids may be subject to varying degrees of isotopic discrimination due to the various biochemical synthetic pathways for amino acids^74^. These different pathways result in some amino acids exhibiting greater isotopic discrimination than others. The difference between the δ^13^C_lipid-free_ values of walrus skin and bone collagen compared with those of liver and muscle likely results from variation in the amino acid composition of these tissues. Walrus skin contains an unusually high proportion of collagen fibrils^71^, making it especially tough. Skin and bone collagen share similar chemical compositions and both contain a large amount of glycine^75^. This amino acid tends to be enriched in ^13^C by ~8.0‰ compared with other amino acids and, as a result, collagen typically has higher δ^13^C values than other tissues^76^. This is likely why, in the present study, walrus skin and bone collagen δ^13^C_lipid-free_ values were similar to one another, and were greater than those of muscle and liver by around 2.0 (95% CI: 1.8–2.2)‰ (Table 5). For δ^15^N, some amino acids exhibit strong ^15^N-enrichment with each trophic transfer (‘trophic amino acids’), whereas others do not (‘source amino acids’)^77^. Walrus skin δ^15^N values were 2.5 (95% CI: 2.3–2.7)‰ greater than those of bone collagen, indicating important differences with regards to discrimination of nitrogen isotopes. In humans, skin collagen has greater proportions of the trophic amino acids alanine, asparagine, and glutamine than bone collagen^75^. This difference may be responsible for the high δ^15^N values of walrus skin in this study, though this could also be due in part to dietary changes and differences in tissue turnover rates.

Quantifying diet-tissue discrimination is important for reconstructing animal diet and understanding food web structure. The results of this study provide information about the relative degree of isotope discrimination among tissues, but their relationship to walrus diet remains unknown. Estimates from other marine mammals indicate an average diet-muscle δ^13^C discrimination factor (Δ^13^C) of +1.2‰ (Supplementary Table 2), with little variation among species (range: +1.0–1.3‰). For δ^15^N, marine mammals average an estimated discrimination factor (Δ^15^N) of +2.8‰ between diet and muscle; however the range of these estimates is much greater (range: +1.7–4.3‰). A rough estimate of diet-muscle discrimination can be made for walruses using published prey stable isotope values. Average reported δ^13^C and δ^15^N of bivalve species common in walrus diet (*Serripes groenlandicus* and *Macoma calcarea*) are ~−18.8‰ and ~9.2‰, respectively^48,49,78,79^. Using these values to estimate diet-muscle discrimination estimates for walruses in this study results in a value of +1.8‰ (Δ^13^C) and +3.3‰ (Δ^15^N). Though bivalves comprise a large portion of their diet, walruses consume a wide variety of prey items, including higher trophic level prey such as predatory snails, seabirds, and seals^49,59,80^. The estimate for Δ^15^N_muscle-diet_ may thus be high, as inclusion of these higher trophic level prey items would increase the estimated average δ^15^N of walrus prey. These estimates are generated from a limited number of prey items, collected in different years than this study’s specimens, so these values are approximations and should be interpreted with caution. Further research is warranted to more precisely estimate diet-tissue discrimination for walruses.

## Conclusions

Stable isotopic analysis of walrus tissues indicates considerable differences among the δ^13^C_lipid-free_ and δ^15^N of muscle, liver, skin, and bone collagen. The differences in δ^13^C_lipid-free_ and δ^15^N among tissues observed in this study generally agree with published values for mammals, with the exception of skin, which differed more substantially from muscle for both δ^13^C_lipid-free_ and δ^15^N. Lipid normalization models were parameterized for walrus muscle and skin. Walrus liver Δδ^13^C was not related to C:N_bulk_, so a mathematical lipid correction could not be generated. Both lipid normalization models performed well, and can be used in future studies as an alternative to chemical lipid extractions. Many walrus muscle samples analyzed in this study had a C:N_bulk_ that indicated they were composed primarily of lean protein (≤3.4) and did not require chemical lipid extraction or mathematical lipid extraction.

The results of the present research will allow for better comparison of δ^13^C and δ^15^N in walrus tissues, giving researchers more flexibility in stable isotope studies of subsistence harvested walruses. Our results will also make studies of δ^13^C and δ^15^N using non-lethal sample collection (i.e., remote skin biopsy) a more viable approach, by decreasing the costs of analysis and providing context for interpreting the results. Finally, the results of this study will be particularly useful for comparing contemporary studies, which typically analyze soft tissues, with historic and paleoecological studies, which generally measure δ^13^C and δ^15^N in bone collagen^23^. The δ^13^C values of walrus skin and bone collagen were indistinguishable, indicating that these tissues likely both primarily represent dietary protein and are suitably comparable. Skin δ^15^N was 2.5 (95% CI: 2.3–2.7)‰ greater than that of bone collagen on average, suggesting differences in nitrogen isotope discrimination between these tissues or a change in diet. The results of this study will also help refine stable isotope mixing models and provide better estimates of walrus diet. Future research measuring the degree of trophic discrimination between the δ^13^C and δ^15^N values of walrus prey and tissues will be important for further improving these reconstructions, as well as for a better understanding of Arctic food web structure and energy flow.

### Ethical approval

This article does not contain any studies with human participants or animals performed by any of the authors.

## Supplementary information

Supplementary Information
