# Author Correction: Lipid normalization and stable isotope discrimination in Pacific walrus tissues

**DOI:** 10.1038/s41598-020-57689-1

**Published:** 2020-01-17

**Authors:** Casey T. Clark, Lara Horstmann, Nicole Misarti

**Affiliations:** 1 Water and Environmental Research Center, University of Alaska Fairbanks, 1764 Tanana Loop, Fairbanks, Alaska 99775-5860 USA; 2 College of Fisheries and Ocean Sciences, University of Alaska Fairbanks, 2150 Koyukuk Drive, Fairbanks, Alaska 99775-7220 USA

Correction to: *Scientific Reports* 10.1038/s41598-019-42095-z, published online 10 April 2019

In the Supplementary Information file originally published with this Article, the University of Alaska Museum specimen IDs were omitted from Supplementary Datasets 1-4.

In addition, the original version of Table 2 contained an error in the Parameters column for the Linear model where,

“*b* = 1.19 (1.02; 1.35)”

now reads:

“*a* = 1.19 (1.02; 1.35)”

These errors have now been corrected in PDF and HTML versions of the Article, and in the accompanying Supplementary Information file.

