# Supplementary material for: Lipid normalization and stable isotope discrimination in Pacific walrus tissues
Source: Sci Rep. 2019 Apr 10;9:5843. doi: 10.1038/s41598-019-42095-z (PMC6458160; doi:10.1038/s41598-019-42095-z)
Supplement: Supplementary file 1 — Supplementary Information [file 41598_2019_42095_MOESM1_ESM.pdf]

Lipid normalization and stable isotope discrimination in Pacific walrus tissues

Casey T. Clark[1,2*], Lara Horstmann[2], Nicole Misarti[1]

[1]Water and Environmental Research Center, University of Alaska Fairbanks, 1764 Tanana Loop, Fairbanks, Alaska 99775-5860, USA

[2]College of Fisheries and Ocean Sciences, University of Alaska Fairbanks, 2150 Koyukuk Drive, Fairbanks, Alaska 99775-7220, USA

*Corresponding author
P.O. Box 757220, Fairbanks, AK 99775-7220, USA
ctclark@alaska.edu

Supplementary Discussion 1:

Female walrus tissues were only available for 2016, and only 10 of the 30 individuals sampled for this study were female. For these reasons, and because there is no evidence to support the assumption that observed isotopic differences in male and female walrus tissues collected in 2016 will remain constant across seasons, years, and longer timespans, sex was not included as a term in the linear mixed effects models presented in the main text of this paper. Visual analysis of the data, however, revealed that there were consistent $\delta^{13}C_{lipid\text{-}free}$ and $\delta^{15}N$ differences between males and females for all tissues examined in this study (Supplementary Discussion Fig. 1). In light of this observation, additional linear mixed effects models were run, including models with terms for sex and an interaction between sex and tissue. Model fit was examined using Akaike's Information Criterion corrected for sample size (AIC_c)[1], as calculated using the AICcmodavg R package[2]. Models were fitted using the R package lme4[3], and *post hoc* pairwise comparison tests were conducted in the lsmeans package in R[4].

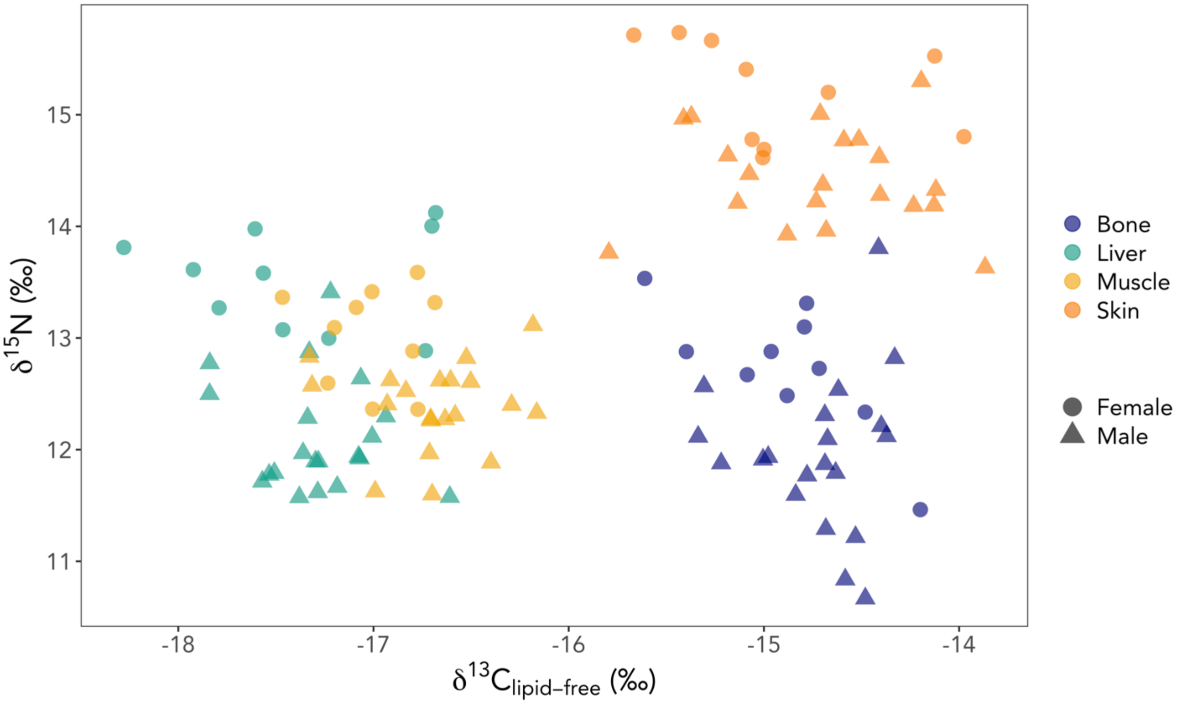

Supplementary Discussion Fig. 1) Scatterplot of $\delta^{13}C_{lipid\text{-}free}$ and $\delta^{15}N$ for bone (blue), liver (green), muscle (yellow), and skin (orange) from female (circles) and male (triangles) Pacific walruses.

The best fitting model for $\delta^{13}C_{lipid\text{-}free}$ included terms for tissue and sex (Supplementary Discussion Table 1). The output from this model indicated that female $\delta^{13}C_{lipid\text{-}free}$ values were 0.2 (95% confidence interval [CI]: 0.0 – 0.4) ‰ lower than those of males for all tissues (Supplementary Discussion Table 2). This value is right on the edge of instrumental error (and its confidence interval extends into the range below instrument error), but is consistent across the four tissues examined in this study and likely represents a real difference. For $\delta^{15}N$, the best fitting model included terms for tissue, sex, and an interaction between tissue and sex (Supplementary Discussion Table 1). Male tissue $\delta^{15}N$ values were consistently lower than those

of females (Supplementary Discussion Tables 3). The interaction between sex and tissue appeared to be driven primarily by liver, for which the differences in male and female $\delta^{15}N$ were nearly double those observed in muscle, skin, and bone (Supplementary Discussion Tables 3 & 4).

Supplementary Discussion Table 1) Model selection results for linear mixed effects models testing the effects of tissue type and sex on $\delta^{13}C_{lipid\text{-}free}$ and $\delta^{15}N$ in walrus tissues. Model formula, number of parameters (k), and ΔAIC_c are presented for each model. All models included a random intercept for individual, to account for correlation among stable isotope values for tissues from individual walruses.

	**$\delta^{13}C_{lipid\text{-}free}$**			**$\delta^{15}N$**	
**Model**	**k**	**ΔAIC_c**	**Model**	**k**	**ΔAIC_c**
~ tissue + sex	7	0.00	~ tissue × sex	10	0.00
~ tissue	6	2.14	~ tissue + sex	7	8.90
~ tissue × sex	10	5.47	~ tissue	6	32.46
Null	3	278.47	Null	3	219.78

Supplementary Discussion Table 2) Coefficient estimates and standard errors for the top-performing linear mixed effects model for $\delta^{13}C_{lipid\text{-}free}$, which included terms for tissue and sex. Coefficient estimates and standard errors for each tissue (muscle, liver, skin, and bone) are presented for females (left two columns) and males (middle two columns). Estimates and 95 % confidence intervals (95 % CI) for the differences between males and females for each tissue are also presented (right two columns). This model also included a random intercept for individual, to account for correlation among stable isotope values for tissues from individual walruses.

	**Female $\delta^{13}C_{lipid\text{-}free}$ (‰)**		**Male $\delta^{13}C_{lipid\text{-}free}$ (‰)**		**Female – Male $\delta^{13}C_{lipid\text{-}free}$ (‰)**	
	Estimate	Std. Error	Estimate	Std. Error	Estimate	95 % CI
**Muscle**	-16.9	0.1	-16.7	0.1	0.2	0.0 – 0.4
**Liver**	-17.5	0.1	-17.3	0.1	0.2	0.0 – 0.4
**Skin**	-14.9	0.1	-14.7	0.1	0.2	0.0 – 0.4
**Bone**	-14.9	0.1	-14.7	0.1	0.2	0.0 – 0.4

Supplementary Discussion Table 3) Coefficient estimates and standard errors for the top-performing linear mixed effects model for $\delta^{15}N$, which included terms for tissue and sex, and an interaction between tissue and sex. Coefficient estimates and standard errors for each tissue (muscle, liver, skin, and bone) are presented for females (left two columns) and males (right two columns). This model also included a random intercept for individual, to account for correlation among stable isotope values for tissues from individual walruses.

	**Female $\delta^{15}N$ (‰)**		**Male $\delta^{15}N$ (‰)**	
	Estimate	Standard Error	Estimate	Standard Error
**Muscle**	13.0	0.2	12.1	0.2
**Liver**	13.5	0.2	11.3	0.2
**Skin**	15.2	0.2	12.0	0.2
**Bone**	12.7	0.2	12.0	0.2

Supplementary Discussion Table 4) Mean differences (95 % confidence intervals) in tissue $\delta^{15}N$ for female (top, white cells) and male (bottom, gray cells) walruses, as estimated by the linear mixed effects models and *post hoc* tests. Bolded values indicate significant differences ($p < 0.05$).

	**Female $\delta^{15}N$ (‰)**				
	**Muscle**	**Liver**	**Skin**	**Bone**	**Female – Male $\delta^{15}N$ (‰)**
**Muscle**	-	0.5 (0.3 – 0.7)	**2.2 (1.8 – 2.6)**	0.3 (0.1 – 0.5)	**0.7 (0.3 – 1.1)**
**Liver**	0.3 (0.1 – 0.5)	-	**1.7 (1.3 – 2.1)**	**0.8 (0.4 – 1.2)**	**1.4 (1.0 – 1.8)**
**Skin**	**2.1 (1.9 – 2.3)**	**2.3 (2.1 – 2.5)**	-	**2.5 (2.1 – 2.9)**	**0.8 (0.4 – 1.2)**
**Bone**	**0.4 (0.2 – 0.6)**	0.2 (0.0 – 0.4)	**2.5 (2.3 – 2.7)**	-	**0.8 (0.4 – 1.2)**
	**Male $\delta^{15}N$ (‰)**				

Male and female walruses exhibit sex-segregated migration[5]. The entire population winters together in the Bering Sea. In the summer, females and juveniles move northward into the Chukchi Sea, whereas most males move in the coastal waters of the Bering Sea, including Bristol Bay and the Russian coastline. The spatial segregation of male and female walruses for much of the year is likely the major driver of observed differences in $\delta^{13}C_{lipid\text{-}free}$ and $\delta^{15}N$ in tissues of males and females. Liver has the fastest turnover rate of the tissues examined[6–9], thus the large difference between male and female liver $\delta^{15}N$ as compared with other tissues analyzed in this study is likely a result of these differences in turnover rate. This is somewhat unexpected, as these animals were harvested in May and June, shortly after males and females had begun to separate and move into different habitats for the summer[5], and both sexes are believed to consume generally the same diet when in the same location[10]. Thus, the large difference in male and female liver $\delta^{15}N$ possibly indicates that the turnover rate of this tissue is slower than expected and liver isotope values still contain a dietary signal reflecting the previous summer when males and females were foraging in different habitats. Alternatively, it might mean that male and female walruses were foraging on different prey while using the same habitat, despite the results of previous studies indicating the contrary. Finally, the difference in liver $\delta^{15}N$ may reflect some other metabolic processes (perhaps associated with fasting or reproductive activities) occurring during the winter months that differ among males and females.

Future research using the tissue-specific discrimination factors presented in this paper could apply the sex-specific discrimination factors in this supplement to known sex animals; however, this should be done with caution. The analyses that examined stable isotope differences between males and females were not included in the main body of the paper because there are a number of caveats that complicate their interpretation. First, there is no reason to believe that the differences between males and females observed in this study will remain constant through time. Second, female tissues were not available in all years, and females made up only 10 of the 30 animals used in this study. Finally, sex may not be available for many historic or archaeological specimens, thus sex-specific discrimination factors are not useful for these samples. For these reasons, it may be more prudent to apply the tissue-specific discrimination factors presented in the main text, rather than the sex-specific discrimination factors presented in this supplement to make corrections among tissues.

References:

1. Burnham, K. P. & Anderson, D. R. *Model selection and multimodel inference: a practical information-theoretic approach*. (Springer Science & Business Media, 2003).
2. Mazerolle, M. J. & Mazerolle, M. M. J. Package 'AICcmodavg'. *R Packag.* (2017).
3. Bates, D., Mächler, M., Bolker, B. M. & Walker, S. C. Fitting linear mixed-effects models using lme4. *J. Stat. Softw.* **67**, 1–48 (2015).
4. Lenth, R. V & others. Least-squares means: the R package lsmeans. *J. Stat. Softw.* **69**, 1–33 (2016).
5. Fay, F. H. Ecology and biology of the Pacific walrus, *Odobenus rosmarus divergens* Illiger. *North Am. Fauna* 1–279 (1982).
6. Tieszen, L. L., Boutton, T. W., Tesdahl, K. G. & Slade, N. A. Fractionation and turnover of stable carbon isotopes in animal tissues: Implications for $\delta^{13}C$ analysis of diet. *Oecologia* **57**, 32–37 (1983).
7. Hobson, K. a & Clark, R. G. Assessing avian diets using stable isotopes II: Factors influencing diet-tissue fractionation. *Condor* **94**, 189–197 (1992).
8. Boecklen, W. J., Yarnes, C. T., Cook, B. A. & James, A. C. On the use of stable isotopes in trophic ecology. *Annu. Rev. Ecol. Evol. Syst.* **42**, 411–440 (2011).
9. Miller, J. F., Millar, J. S. & Longstaffe, F. J. Carbon- and nitrogen-isotope tissue–diet discrimination and turnover rates in deer mice, *Peromyscus maniculatus*. *Can. J. Zool.* **86**, 685–691 (2008).
10. Sheffield, G. & Grebmeier, J. M. Pacific walrus (*Odobenus rosmarus divergens*): Differential prey digestion and diet. *Mar. Mammal Sci.* **25**, 761–777 (2009).

Supplementary Table 1. Examples of studies using lipid normalization models to estimate $\delta^{13}C_{lipid\text{-}free}$ in the tissues of invertebrates, fishes, amphibians, reptiles, birds, and mammals.

Taxon	Example References
Invertebrates	Kiljunen et al. (2006); Bodin et al. (2007); Logan et al. (2008); de Lecea and de Charmoy (2015); Choy et al. (2016)
Fishes	Sweeting et al. (2006); Kiljunen et al. (2006); Logan et al. (2008); Hoffman and Sutton (2010); Fagan et al. (2011); Reum (2011); Abrantes et al. (2012); de Lecea and de Charmoy (2015); Hoffman et al. (2015); Sardenne et al. (2015); Skinner et al. (2016); Giménez et al. (2017)
Amphibians	Trakimas et al. (2011); Caut et al. (2013); Arribas et al. (2015)
Reptiles	Rosenblatt and Heithaus (2013); Medeiros et al. (2015)
Birds	Kojadinovic et al. (2008); Doucette et al. (2010); Ehrich et al. (2011); Elliott et al. (2014); Elliott and Elliott (2016)
Mammals	Ehrich et al. (2011); Ryan et al. (2012); Yurkowski et al. (2015); Choy et al. (2016); Giménez et al. (2017)

Supplementary Table 2) Estimates of mammalian tissue-specific isotopic discrimination factors from this study and from the published literature. Diet-muscle fractionation of $\delta^{13}C$ and $\delta^{15}N$ is included where available, and values for all other tissues (liver, skin, bone collagen, hair/feather, whole blood, red blood cells) are expressed as the difference (‰) in $\delta^{13}C$ and $\delta^{15}N$ between each tissue and muscle (tissue – muscle).

**Source**	**Species**	**Muscle − Diet**		**Liver − Muscle**		**Skin − Muscle**		**Bone Collagen − Muscle**		**Hair/Feather − Muscle**	
		**Δ $\delta^{13}C$ (‰)**	**Δ $\delta^{15}N$ (‰)**	**Δ $\delta^{13}C$ (‰)**	**Δ $\delta^{15}N$ (‰)**	**Δ $\delta^{13}C$ (‰)**	**Δ $\delta^{15}N$ (‰)**	**Δ $\delta^{13}C$ (‰)**	**Δ $\delta^{15}N$ (‰)**	**Δ $\delta^{13}C$ (‰)**	**Δ $\delta^{15}N$ (‰)**
This study	Pacific Walrus *Odobenus rosmarus divergens*	-	-	−0.5	0.0	2.0	2.1	2.0	-0.4	-	-
Pinzone et al. (2017)	Hooded seal *Cystophora cristata*	1.1	4.3	-	-	-	-	-	-	0.8	−0.7
Sinisalo et al. (2008)	Ringed seal *Pusa hispida*	-	-	0.0	0.4	-	-	-	-	-	-
Hobson et al. (1996)	Harp, harbor, ringed seals *Pagophilus groenlandicus, Phoca vitulina, Pusa hispida*	1.3	2.4	−0.7	0.7	1.5	-0.1	-	-	1.5	0.6
Todd et al. (2010)	Steller sea lion *Eumetopias jubatus*	-	-	-	-	0.6	0.2	-	-	-	-
Todd et al. (2010)	California sea lion *Zalophus californianus*	-	-	-	-	0.9	0.5	-	-	-	-
Kurle and Worthy (2002)	Northern fur seal *Callorhinus ursinus*	-	-	−0.2	0.8	-	-	-	-	0.4	0.4
Jansen et al. (2012)	Harbor porpoise *Phocoena phocoena*	-	-	-	-	-	-	2.4	0.0	-	-
Toperoff (2002)	Harbor porpoise *Phocoena phocoena*	1.0	1.7	-	-	0.0	0.8	2.8	0.5	-	-
Borrell et al. (2012)	Fin whale *Balaenoptera physalus*	1.3	2.7	0.4	1.2	0.0	0.1	1.8	-0.7	-	-

Horstmann-Dehn et al. (2012)	Bowhead whale<br>*Balaena mysticetus*	-	-	-	-	−0.2	0.3	-	-	-	-
Horstmann-Dehn et al. (2012)	Gray whale<br>*Eschrichtius robustus*	-	-	-	-	0.6	1.2	-	-	-	-
Horstmann-Dehn et al. (2012)	Beluga<br>*Delphinapterus leucas*	-	-	-	-	−0.4	0.1	-	-	-	-
Abend and Smith (1997)	Long-finned pilot whale<br>*Globicephala melas*	1.1	-	-	-	−0.8	0.5	-	-	-	-
Abend and Smith (1995)	Long-finned pilot whale<br>*Globicephala melas*	-	-	-	-	-	0.6	-	-	-	-
Todd et al. (1997)	Humpback whale<br>*Megaptera novaeangliae*	-	-	-	-	-0.5	-	-	-	-	-
Kurle et al. (2014)	Norway rat<br>*Rattus norvegicus*	1.8	2.6	−0.4	0.5	-	-	-	-	0.4	0.4
Webb et al. (2016)	Domestic pig<br>*Sus domesticus*	-	2.4	-	0.8	-	-	-	0.1	-	-
Warinner and Tuross (2010)	Domestic pig<br>*Sus domesticus*	2.0	2.1	-	-	-	-	1.3	−0.3	1.7	0.3
Nardoto et al. (2006)	Wild boar<br>*Sus scrofa*	−1.6	2.8	−0.8	−0.6	-	-	-	-	1.8	−0.1
Miller et al. (2008)	Deer mouse<br>*Peromyscus maniculatus*	−0.7	2.5	−0.1	0.9	-	-	-	-	1.0	0.8
Arneson and MacAvoy (2005)	House mouse<br>*Mus musculus*	0.2	2.7	−0.3	1.5	-	-	-	-	-	-
MacAvoy et al. (2005)	House mouse<br>*Mus musculus*	2.5	3.3	−0.7	2.0	-	-	-	-	-	-
Roth and Hobson (2000)	Red fox<br>*Vulpes vulpes*	1.1	3.5	−0.7	0.0	-	-	-	-	1.5	−0.2
Tieszen et al. (1983)	Gerbil<br>*Meriones unguiculatus*	−0.2	-	−0.5	-	-	-	-	-	1.3	-

Marine Mammal Mean	1.2	2.8	−0.2	0.6	0.3	0.6	2.3	−0.2	0.9	0.1
1 Standard Deviation	0.1	1.1	0.4	0.4	0.9	0.6	0.4	0.5	0.6	0.7
All Mammal Mean	0.8	2.8	−0.4	0.7	0.3	0.6	2.1	−0.1	1.2	0.2
1 Standard Deviation	1.1	0.7	0.4	0.7	0.9	0.6	0.6	0.4	0.5	0.5

Abend, A. G., and T. D. Smith. 1995. Differences in ratios of stable isotopes of nitrogen in long-finned pilot whales (*Globicephala melas*) in the western and eastern North Atlantic. ICES Journal of Marine Science **52**: 837–841.

Abend, A. G., and T. D. Smith. 1997. Differences in stable isotope ratios of carbon and nitrogen between long-finned pilot whales (*Globicephala melas*) and their primary prey in the western north Atlantic. ICES Journal of Marine Science **54**: 500–503.

Abrantes, K. G., J. M. Semmens, J. M. Lyle, and P. D. Nichols. 2012. Normalisation models for accounting for fat content in stable isotope measurements in salmonid muscle tissue. Marine Biology **159**: 57–64. doi:10.1007/s00227-011-1789-1

Arneson, L. S., and S. E. MacAvoy. 2005. Carbon, nitrogen, and sulfur diet-tissue discrimination in mouse tissues. Canadian Journal of Zoology **83**: 989–995. doi:10.1139/Z05-083

Arribas, R., C. Díaz-Paniagua, S. Caut, and I. Gomez-Mestre. 2015. Stable isotopes reveal trophic partitioning and trophic plasticity of a larval amphibian guild. PLoS ONE **10**: 1–19. doi:10.1371/journal.pone.0130897

Bodin, N., F. Le Loc'h, and C. Hily. 2007. Effect of lipid removal on carbon and nitrogen stable isotope ratios in crustacean tissues. Journal of Experimental Marine Biology and Ecology **341**: 168–175. doi:10.1016/j.jembe.2006.09.008

Borrell, A., N. Abad-Oliva, E. Gõmez-Campos, J. Giménez, and A. Aguilar. 2012. Discrimination of stable isotopes in fin whale tissues and application to diet assessment in cetaceans. Rapid Communications in Mass Spectrometry **26**: 1596–1602. doi:10.1002/rcm.6267

Caut, S., E. Angulo, C. Díaz-Paniagua, and I. Gomez-Mestre. 2013. Plastic changes in tadpole trophic ecology revealed by stable isotope analysis. Oecologia **173**: 95–105. doi:10.1007/s00442-012-2428-3

Choy, E. S., J. D. Roth, and L. L. Loseto. 2016. Lipid removal and acidification affect nitrogen and carbon stable isotope ratios of beluga whales (*Delphinapterus leucas*) and their potential prey species in the Beaufort Sea ecosystem. Marine Biology **163**: 1–13. doi:10.1007/s00227-016-2992-x

Doucette, J. L., B. Wissel, and C. M. Somers. 2010. Effects of lipid extraction and lipid normalization on stable carbon and nitrogen isotope ratios in double-crested cormorants: Implications for food web studies. Waterbirds **33**: 273–284. doi:10.1675/063.033.0302

Ehrich, D., A. Tarroux, J. Stien, N. Lecomte, S. Killengreen, D. Berteaux, and N. G. Yoccoz. 2011. Stable isotope analysis: Modelling lipid normalization for muscle and eggs from arctic mammals and birds. Methods in Ecology and Evolution **2**: 66–76. doi:10.1111/j.2041-210X.2010.00047.x

Elliott, K. H., M. Davis, and J. E. Elliott. 2014. Equations for lipid normalization of carbon stable isotope ratios in aquatic bird eggs. PLoS ONE **9**: 33–36. doi:10.1371/journal.pone.0083597

Elliott, K. H., and J. E. Elliott. 2016. Lipid extraction techniques for stable isotope analysis of bird eggs: Chloroform-methanol leads to more enriched ^{13}C values than extraction via

petroleum ether. Journal of Experimental Marine Biology and Ecology **474**: 54–57. doi:10.1016/j.jembe.2015.09.017

Fagan, K.-A., M. A. Koops, M. T. Arts, and M. Power. 2011. Assessing the utility of C:N ratios for predicting lipid content in fishes. Canadian Journal of Fisheries and Aquatic Sciences **68**: 374–385. doi:10.1139/F10-119

Giménez, J., F. Ramírez, M. G. Forero, J. Almunia, R. de Stephanis, and J. Navarro. 2017. Lipid effects on isotopic values in bottlenose dolphins (*Tursiops truncatus*) and their prey with implications for diet assessment. Marine Biology **164**: 1–9. doi:10.1007/s00227-017-3154-5

Hobson, K. A., D. M. Schell, D. Renouf, and E. Noseworthy. 1996. Stable carbon and nitrogen isotopic fractionation between diet and tissues of captive seals: implications for dietary reconstructions involving marine mammals. Canadian Journal of Fisheries and Aquatic Sciences **53**: 528–533.

Hoffman, J. C., M. E. Sierszen, and A. M. Cotter. 2015. Fish tissue lipid-C:N relationships for correcting $\delta^{13}C$ values and estimating lipid content in aquatic food-web studies. Rapid Communications in Mass Spectrometry **29**: 2069–2077. doi:10.1002/rcm.7367

Hoffman, J. C., and T. T. Sutton. 2010. Lipid correction for carbon stable isotope analysis of deep-sea fishes. Deep-Sea Research Part I: Oceanographic Research Papers **57**: 956–964. doi:10.1016/j.dsr.2010.05.003

Horstmann-Dehn, L., E. H. Follmann, C. Rosa, G. Zelensky, and C. George. 2012. Stable carbon and nitrogen isotope ratios in muscle and epidermis of arctic whales. Marine Mammal Science **28**: E173–E190. doi:10.1111/j.1748-7692.2011.00503.x

Jansen, O. E., G. M. Aarts, K. Das, and others. 2012. Feeding ecology of harbour porpoises: stable isotope analysis of carbon and nitrogen in muscle and bone. Marine Biology Research **8**: 829–841. doi:10.1080/17451000.2012.692164

Kiljunen, M., J. Grey, T. Sinisalo, C. Harrod, H. Immonen, and R. I. Jones. 2006. A revised model for lipid-normalizing $\delta^{13}C$ values from aquatic organisms, with implications for isotope mixing models. Journal of Applied Ecology **43**: 1213–1222. doi:10.1111/j.1365-2664.2006.01224.x

Kojadinovic, J., P. Richard, M. Le Corre, R. P. Cosson, and P. Bustamante. 2008. Effects of lipid extraction on $\delta^{13}C$ and $\delta^{15}N$ values in seabird muscle, liver and feathers. Waterbirds **31**: 169–178. doi:10.1675/1524-4695(2008)31[169:EOLEOC]2.0.CO;2

Kurle, C. M., P. L. Koch, B. R. Tershy, and D. A. Croll. 2014. The effects of sex, tissue type, and dietary components on stable isotope discrimination factors ($\Delta^{13}C$ and $\Delta^{15}N$) in mammalian omnivores. Isotopes in Environmental and Health Studies **50**: 307–321. doi:10.1080/10256016.2014.908872

Kurle, C. M., and G. A. J. Worthy. 2002. Stable nitrogen and carbon isotope ratios in multiple tissues of the northern fur seal *Callorhinus ursinus*: implications for dietary and migratory reconstructions. Marine Ecology Progress Series **236**: 289–300.

de Lecea, A. M., and L. de Charmoy. 2015. Chemical lipid extraction or mathematical isotope

correction models: Should mathematical models be widely applied to marine species? Rapid Communications in Mass Spectrometry **29**: 2013–2025. doi:10.1002/rcm.7310

Logan, J. M., T. D. Jardine, T. J. Miller, S. E. Bunn, R. A. Cunjak, and M. E. Lutcavage. 2008. Lipid corrections in carbon and nitrogen stable isotope analyses: Comparison of chemical extraction and modelling methods. Journal of Animal Ecology **77**: 838–846. doi:10.1111/j.1365-2656.2008.01394.x

MacAvoy, S. E., S. A. Macko, and L. S. Arneson. 2005. Growth versus metabolic tissue replacement in mouse tissues determined by stable carbon and nitrogen isotope analysis. Canadian Journal of Zoology **83**: 631–641. doi:10.1139/z05-038

Medeiros, L., D. da Silveira Monteiro, R. Petitet, and L. Bugoni. 2015. Effects of lipid extraction on the isotopic values of sea turtle bone collagen. Aquatic Biology **23**: 191–199. doi:10.3354/ab00628

Miller, J. F., J. S. Millar, and F. J. Longstaffe. 2008. Carbon- and nitrogen-isotope tissue–diet discrimination and turnover rates in deer mice, *Peromyscus maniculatus*. Canadian Journal of Zoology **86**: 685–691. doi:10.1139/Z08-042

Nardoto, G. B., S. Silva, C. Kendall, and others. 2006. Geographical patterns of human diet derived from stable-isotope analysis of fingernails. American Journal of Physical Anthropology **131**: 137–146. doi:10.1002/ajpa.20409

Pinzone, M., L. N. Michel, M. Acquarone, L. Huyghebaert, N. Sturaro, U. Siebert, and K. Das. 2017. Carbon, nitrogen and sulphur isotopic fractionation in captive juvenile hooded seal (*Cystophora cristata*): Application for diet analysis. Rapid Communications in Mass Spectrometry **31**: 1720–1728. doi:10.1002/rcm.7955

Reum, J. C. P. 2011. Lipid correction model of carbon stable isotopes for a cosmopolitan predator, spiny dogfish *Squalus acanthias*. Journal of Fish Biology **79**: 2060–2066. doi:10.1111/j.1095-8649.2011.03120.x

Rosenblatt, A. E., and M. R. Heithaus. 2013. Slow isotope turnover rates and low discrimination values in the American alligator: Implications for interpretation of ectotherm stable isotope data. Physiological and Biochemical Zoology **86**: 137–148. doi:10.1086/668295

Roth, J. D., and K. A. Hobson. 2000. Stable carbon and nitrogen isotopic fractionation between diet and tissue of captive red fox: implications for dietary reconstruction. Canadian Journal of Zoology **78**: 848–852.

Ryan, C., B. McHugh, C. N. Trueman, C. Harrod, S. D. Berrow, and I. O'Connor. 2012. Accounting for the effects of lipids in stable isotope ($\delta^{13}C$ and $\delta^{15}N$ values) analysis of skin and blubber of balaenopterid whales. Rapid Communications in Mass Spectrometry **26**: 2745–2754. doi:10.1002/rcm.6394

Sardenne, F., F. Ménard, M. Degroote, E. Fouché, G. Guillou, B. Lebreton, S. J. Hollanda, and N. Bodin. 2015. Methods of lipid-normalization for multi-tissue stable isotope analyses in tropical tuna. Rapid Communications in Mass Spectrometry **29**: 1253–1267. doi:10.1002/rcm.7215

Sinisalo, T., R. I. Jones, E. Helle, and E. T. Valtonen. 2008. Changes in diets of individual Baltic ringed seals (*Phoca hispida botnica*) during their breeding season inferred from stable isotope analysis of multiple tissues. Marine Mammal Science **24**: 159–170. doi:10.1111/j.1748-7692.2007.00170.x

Skinner, M. M., A. A. Martin, and B. C. Moore. 2016. Is lipid correction necessary in the stable isotope analysis of fish tissues? Rapid Communications in Mass Spectrometry **30**: 881–889. doi:10.1002/rcm.7480

Sweeting, C. J., N. V. C. Polunin, and S. Jennings. 2006. Effects of chemical lipid extraction and arithmetic lipid correction on stable isotope ratios of fish tissues. Rapid Communications in Mass Spectrometry **20**: 595–601. doi:10.1002/rcm.2347

Tieszen, L. L., T. W. Boutton, K. G. Tesdahl, and N. A. Slade. 1983. Fractionation and turnover of stable carbon isotopes in animal tissues: Implications for $\delta^{13}C$ analysis of diet. Oecologia **57**: 32–37. doi:10.1007/BF00379558

Todd, S. K., B. Holm, D. A. S. Rosen, and D. J. Tollit. 2010. Stable isotope signal homogeneity and differences between and within pinniped muscle and skin. Marine Mammal Science **26**: 176–185. doi:10.1111/j.1748-7692.2009.00345.x

Todd, S., P. Ostrom, J. Lien, and J. Abrajano. 1997. Use of biopsy samples of humpback whale (*Megaptera novaeangliae*) skin for stable isotope ($\delta^{13}C$) determination. Journal Of Northwest Atlantic Fishery Science **22**: 71–76. doi:10.2960/J.v22.a6

Toperoff, A. K. 2002. Examination of diet of harbor porpoise (*Phocoena phocoena*) from central California using stomach content and stable isotope analysis from multiple tissues.

Trakimas, G., T. D. Jardine, R. Barisevičiute, A. Garbaras, R. Skipityte, and V. Remeikis. 2011. Ontogenetic dietary shifts in European common frog (*Rana temporaria*) revealed by stable isotopes. Hydrobiologia **675**: 87–95. doi:10.1007/s10750-011-0804-3

Warinner, C., and N. Tuross. 2010. Tissue isotopic enrichment associated with growth depression in a pig: Implications for archaeology and ecology. American Journal of Physical Anthropology **141**: 486–493. doi:10.1002/ajpa.21222

Webb, E. C., A. Stewart, B. Miller, J. Tarlton, and R. P. Evershed. 2016. Age effects and the influence of varying proportions of terrestrial and marine dietary protein on the stable nitrogen-isotope compositions of pig bone collagen and soft tissues from a controlled feeding experiment. STAR: Science & Technology of Archaeological Research **2**: 54–66. doi:10.1080/20548923.2015.1133121

Yurkowski, D. J., N. E. Hussey, C. Semeniuk, S. H. Ferguson, and A. T. Fisk. 2015. Effects of lipid extraction and the utility of lipid normalization models on $\delta^{13}C$ and $\delta^{15}N$ values in Arctic marine mammal tissues. Polar Biology **38**: 131–143. doi:10.1007/s00300-014-1571-1

Supplementary Dataset 1. Raw stable isotope data for walrus bone collagen samples including animal ID, University of Alaska Museum specimen ID (UAM ID), sex, $\delta^{15}N$ (‰), $\delta^{13}C$ (‰), and carbon:nitrogen ratio. Asterisks next to IDs indicate individuals with all tissues (muscle, liver, skin, and bone collagen) used to evaluate tissue-specific isotope discrimination. Lipid-free values are not provided because collagen is a purified protein and does not require lipid extraction.

BONE COLLAGEN

ID	UAM ID	Sex	$\delta^{15}N$ (‰)	$\delta^{13}C$ (‰)	C:N
WAL137*	125312	M	11.9	-14.7	2.8
WAL146*	129168	M	10.8	-14.6	2.8
WAL234*	-	M	11.6	-14.8	2.7
WAL235*	-	M	12.1	-14.4	2.8
WAL238*	131795	M	11.3	-14.7	2.8
WAL239*	-	M	10.7	-14.5	2.8
WAL240*	-	M	12.6	-15.3	2.8
WAL779*	-	F	13.5	-15.6	2.9
WAL780*	131823	F	12.9	-15.0	2.8
WAL781*	131819	F	13.3	-14.8	2.7
WAL783*	131817	F	12.9	-15.4	2.8
WAL785*	131820	F	12.7	-14.7	2.9
WAL786*	131816	F	13.1	-14.8	3.0
WAL788*	131818	F	11.5	-14.2	2.9
WAL790*	131815	F	12.3	-14.5	2.8
WAL791*	131799	M	12.8	-14.3	2.8
WAL792*	131813	F	12.5	-14.9	3.0
WAL794*	131796	M	11.9	-15.0	2.9
WAL795*	131803	M	11.8	-14.8	2.9
WAL796*	131808	M	13.8	-14.4	2.8
WAL797*	131806	M	12.1	-15.3	2.9
WAL798*	131800	M	11.9	-15.2	3.0
WAL799*	131802	M	12.1	-14.7	3.0
WAL800*	131809	M	11.2	-14.5	2.9
WAL802*	131801	M	11.9	-15.0	3.1
WAL803*	131798	F	12.7	-15.1	3.0
WAL805*	131811	M	12.3	-14.7	2.9
WAL806*	131797	M	11.8	-14.6	2.9
WAL807*	131804	M	12.2	-14.4	3.0
WAL808*	131810	M	12.5	-14.6	2.9

Supplementary Dataset 2. Raw stable isotope data for walrus muscle samples including animal ID, University of Alaska Museum specimen ID (UAM ID), sex, $\delta^{15}N$ (‰), $\delta^{13}C$ (‰), C:N, $\delta^{15}N_{lipid\text{-}free}$ (‰), $\delta^{13}C_{lipid\text{-}free}$ (‰), $C:N_{lipid\text{-}free}$. Asterisks next to IDs indicate individuals with all tissues (muscle, liver, skin, and bone collagen) used to evaluate tissue-specific isotope discrimination.

MUSCLE

ID	UAM ID	Sex	$\delta^{15}N$ (‰)	$\delta^{13}C$ (‰)	C:N	$\delta^{15}N_{lipid\text{-}free}$ (‰)	$\delta^{13}C_{lipid\text{-}free}$ (‰)	$C:N_{lipid\text{-}free}$
WAL125	125300	M	13.6	−16.5	3.3	13.5	−17.2	3.1
WAL128	125302	M	12.3	−16.3	3.4	12.3	−16.2	3.3
WAL129	125303	M	13.2	−17.0	3.3	13.8	−16.8	3.2
WAL132	125306	F	12.8	−16.0	3.8	13.8	−15.2	3.2
WAL133	125307	M	11.8	−17.2	3.4	12.5	−16.0	3.1
WAL134	125309	M	12.5	−17.5	3.7	13.4	−16.3	3.2
WAL135	125310	M	12.2	−17.6	3.7	12.7	−17.0	3.3
WAL137*	125312	M	13.0	−16.4	3.2	13.5	−16.4	3.1
WAL138	125313	M	11.4	−16.9	3.3	11.6	−17.3	3.2
WAL139	125314	M	12.5	−16.9	3.2	12.4	−17.4	3.2
WAL140	125315	F	12.2	−17.1	3.4	12.5	−16.9	3.2
WAL141	125316	M	12.3	−16.4	3.3	12.1	−16.9	3.3
WAL143	125318	M	12.4	−16.3	3.2	12.2	−16.6	3.2
WAL144	125319	M	12.5	−16.7	3.4	12.2	−16.9	3.3
WAL145	125322	M	12.9	−16.8	3.3	12.6	−17.2	3.2
WAL146*	129168	M	12.1	−16.5	3.3	11.9	−17.0	3.2
WAL147	125324	M	12.3	−17.9	3.7	12.1	−17.6	3.3
WAL148	125327	M	12.6	−16.3	3.3	12.4	−16.7	3.2
WAL149	125328	F	13.1	−17.3	3.4	12.9	−17.4	3.3
WAL151	125287	F	12.1	−15.7	3.3	12.5	−15.4	3.2
WAL152	125288	M	12.0	−16.9	3.3	12.1	−17.3	3.2
WAL153	125289	F	12.0	−17.2	3.5	11.5	−17.2	3.3
WAL154	125290	M	12.3	−16.6	3.4	12.2	−17.0	3.2
WAL157	125293	F	12.5	−17.3	3.4	13.0	−17.0	3.2
WAL234*	-	M	12.3	−17.0	3.5	12.2	−16.7	3.1
WAL235*	-	M	12.3	−16.6	3.4	12.5	−16.2	3.1
WAL237	-	M	12.9	−18.0	4.2	13.2	−16.2	3.1
WAL238*	131795	M	12.5	−16.6	3.8	12.7	−16.3	3.6
WAL239*	-	M	12.3	−16.8	3.5	12.2	−16.6	3.1
WAL240*	-	M	12.6	−17.5	3.6	12.6	−17.3	3.2
WAL283	-	M	12.9	−16.9	3.7	12.9	−16.6	3.5
WAL434	116517	M	11.8	−16.0	3.3	11.9	−16.0	3.3
WAL435	-	M	13.7	−17.1	3.4	14.1	−17.5	3.3
WAL436	-	F	12.2	−17.2	3.4	12.4	−17.5	3.3
WAL438	-	M	12.1	−16.6	3.3	12.2	−17.1	3.3
WAL439	-	F	12.2	−19.2	4.8	12.9	−17.6	3.4
WAL443	-	F	12.3	−17.4	3.5	12.7	−17.5	3.3
WAL779*	-	F	13.3	−17.7	3.4	13.4	−17.5	3.4

WAL780*	131823	F	13.6	−16.8	3.4	13.6	−16.8	3.4
WAL781*	131819	F	13.3	−17.3	3.4	13.4	−17.1	3.4
WAL782	131814	F	13.4	−19.0	5.4	13.4	−16.7	3.5
WAL783*	131817	F	13.1	−17.5	3.6	13.1	−17.2	3.5
WAL784	131824	F	12.9	−16.9	3.4	12.7	−16.8	3.4
WAL785*	131820	F	13.3	−17.1	3.5	13.3	−16.7	3.4
WAL786*	131816	F	13.5	−17.8	3.5	13.1	−17.0	3.5
WAL787	131821	F	13.0	−17.3	3.6	12.6	−17.5	3.5
WAL788*	131818	F	12.6	−17.9	3.7	12.5	−17.2	3.5
WAL789	131822	F	12.5	−17.7	3.6	13.5	−17.5	3.5
WAL790*	131815	F	12.4	−17.1	4.0	12.5	−17.0	3.7
WAL791*	131799	M	12.6	−16.7	3.3	12.3	−16.5	3.1
WAL792*	131813	F	12.9	−17.0	3.4	12.9	−16.8	3.2
WAL793	131807	M	12.5	−16.7	3.4	12.3	−16.5	3.2
WAL794*	131796	M	12.6	−16.9	3.4	12.6	−16.7	3.3
WAL795*	131803	M	12.4	−16.9	3.3	12.3	−16.9	3.2
WAL796*	131808	M	12.4	−16.8	3.4	12.4	−16.7	3.3
WAL797*	131806	M	12.8	−16.6	3.4	12.7	−16.5	3.3
WAL798*	131800	M	12.6	−16.8	3.4	12.6	−16.8	3.3
WAL799*	131802	M	12.6	−16.6	3.4	12.5	−16.6	3.3
WAL800*	131809	M	11.6	−16.7	3.4	11.6	−16.7	3.3
WAL802*	131801	M	12.3	−16.6	3.4	12.4	−16.6	3.3
WAL803*	131798	F	12.4	−17.1	3.5	12.5	−16.8	3.4
WAL805*	131811	M	12.0	−16.9	3.6	12.1	−16.7	3.4
WAL806*	131797	M	12.6	−17.2	3.6	12.6	−16.9	3.4
WAL807*	131804	M	12.0	−16.6	3.4	12.1	−16.4	3.3
WAL808*	131810	M	13.1	−16.4	3.5	13.1	−16.2	3.4
WAL949	116807	M	11.7	−17.1	3.5	12.0	−17.3	3.3
WAL950	116808	M	12.4	−16.9	3.5	12.5	−17.2	3.4
WAL951	116811	M	12.0	−17.0	3.4	12.0	−17.4	3.3
WAL952	116530	M	11.8	−16.5	3.3	12.0	−16.9	3.3
WAL953	116716	M	12.1	−17.0	3.4	12.2	−17.4	3.3
WAL954	116717	M	11.9	−17.1	3.4	12.2	−17.4	3.3
WAL955	116614	M	11.6	−16.6	3.3	12.1	−17.1	3.2
WAL956	116718	M	12.3	−16.8	3.3	12.9	−17.3	3.2
WAL957	116711	M	12.1	−16.6	3.4	12.5	−16.7	3.3
WAL958	116509	F	11.7	−17.6	3.6	12.1	−17.5	3.4
WAL959	116876	M	11.4	−17.5	3.5	11.7	−17.8	3.3
WAL960	-	U	13.5	−17.3	3.6	13.9	−17.3	3.3
WAL961	116648	M	12.2	−16.7	3.3	12.5	−16.5	3.2
WAL962	116747	M	12.2	−16.6	3.3	12.5	−16.5	3.2
WAL963	116487	M	12.1	−16.3	3.4	12.4	−16.6	3.2
WAL964	116615	M	14.1	−16.4	3.2	14.2	−16.1	3.1
WAL965	116616	M	12.9	−16.7	3.5	12.5	−17.2	3.3
WAL966	116539	F	14.0	−17.6	3.4	13.9	−17.6	3.4

WAL967	116371	F	13.6	−17.6	3.6	14.0	−17.6	3.3
WAL968	116540	F	12.2	−17.2	3.4	12.2	−17.6	3.3
WAL969	117032	F	12.5	−19.5	5.7	13.1	−17.5	3.3
WAL970	116460	F	13.0	−17.5	3.3	13.2	−17.9	3.2
WAL971	117034	F	12.4	−16.8	3.3	12.5	−17.2	3.3
WAL972	116442	F	13.4	−17.4	3.5	13.5	−17.7	3.3
WAL973	116653	F	13.1	−17.9	3.6	12.9	−18.0	3.3
WAL974	116338	F	12.1	−17.2	3.4	12.4	−17.3	3.3
WAL975	116492	F	12.0	−17.1	3.4	12.1	−17.3	3.4
WAL976	116478	F	13.1	−17.3	3.4	13.6	−17.5	3.4
WAL977	116445	F	11.8	−16.9	3.3	11.9	−17.4	3.3
WAL978	116495	F	11.6	−16.7	3.4	12.0	−16.8	3.6
WAL979	116748	F	11.5	−18.4	4.0	11.9	−17.7	3.4
WAL980	116372	F	12.6	−17.6	3.6	13.0	−17.5	3.5
WAL981	116679	F	13.0	−17.7	3.6	13.9	−17.7	3.3
WAL982	116749	F	12.2	−17.8	3.5	12.7	−17.8	3.3
WAL983	116339	F	12.5	−17.9	3.6	12.8	−17.8	3.3
WAL984	116462	F	12.5	−17.3	3.5	12.7	−16.7	3.2
WAL985	119507	M	12.0	−17.4	3.7	12.1	−16.9	3.3
WAL986	119508	M	12.2	−17.3	3.8	12.1	−16.8	3.3
WAL987	119509	M	12.6	−16.7	3.4	12.5	−16.9	3.3
WAL988	119511	M	12.5	−17.2	3.6	12.5	−17.3	3.4
WAL989	119517	M	12.5	−16.6	3.4	12.2	−17.1	3.3
WAL990	119498	M	12.5	−17.1	3.4	12.1	−17.2	3.2
WAL991	119500	M	12.1	−16.5	3.4	12.1	−16.6	3.3
WAL992	119486	F	13.1	−17.8	3.7	12.4	−17.4	3.3
WAL993	119501	M	12.5	−17.3	3.5	12.3	−17.5	3.3
WAL994	119487	F	12.6	−18.0	3.8	12.3	−17.3	3.2
WAL995	119502	M	12.5	−16.6	3.6	12.3	−16.8	3.4
WAL996	119488	F	13.3	−17.1	3.4	13.4	−17.0	3.2
WAL997	119504	M	12.2	−16.4	3.3	12.0	−16.9	3.2
WAL998	119505	M	13.0	−17.3	3.5	13.1	−17.7	3.3
WAL999	119490	F	12.6	−17.6	3.5	12.6	−17.6	3.3
WAL1000	125308	M	12.0	−16.4	3.4	12.9	−16.1	3.1
WAL1001	125317	M	12.3	−16.6	3.3	12.2	−17.1	3.3
WAL1002	125318	M	12.1	−16.8	3.4	12.0	−17.1	3.3
WAL1003	125321	M	11.8	−16.4	3.3	12.0	−16.4	3.2
WAL1004	125325	M	12.2	−16.4	3.3	12.0	−17.0	3.2
WAL1005	125330	M	12.5	−16.5	3.3	12.3	−16.9	3.3
WAL1006	125332	M	11.3	−17.4	3.5	10.8	−17.4	3.3
WAL1007	125333	M	12.0	−16.2	3.2	11.4	−16.9	3.2
WAL1008	-	M	12.7	−16.8	3.3	12.0	−17.3	3.3

Supplementary Dataset 3. Raw stable isotope data for walrus liver samples including animal ID, University of Alaska Museum specimen ID (UAM ID), sex, $\delta^{15}N$ (‰), $\delta^{13}C$ (‰), C:N, $\delta^{15}N_{lipid\text{-}free}$ (‰), $\delta^{13}C_{lipid\text{-}free}$ (‰), C:$N_{lipid\text{-}free}$. Asterisks next to IDs indicate individuals with all tissues (muscle, liver, skin, and bone collagen) used to evaluate tissue-specific isotope discrimination.

LIVER								
**ID**	**UAM ID**	**Sex**	**$\delta^{15}N$ (‰)**	**$\delta^{13}C$ (‰)**	**C:N**	**$\delta^{15}N_{lipid\text{-}free}$ (‰)**	**$\delta^{13}C_{lipid\text{-}free}$ (‰)**	**C:$N_{lipid\text{-}free}$**
WAL137*	125312	M	12.8	-18.1	4.2	12.9	-17.8	4.1
WAL146*	129168	M	11.9	-17.8	4.8	12.1	-17.1	4.2
WAL234*	-	M	11.9	-18.1	3.9	12.1	-17.3	3.4
WAL235*	-	M	11.8	-18.4	5.0	12.7	-17.5	4.5
WAL238*	131795	M	12.6	-17.9	5.1	12.8	-17.1	4.6
WAL239*	-	M	11.6	-18.2	4.5	12.4	-17.3	4.0
WAL240*	-	M	12.0	-18.3	4.2	12.2	-17.4	3.7
WAL779*	-	F	13.8	-19.4	5.2	14.0	-18.3	4.2
WAL780*	131823	F	14.0	-17.9	4.5	14.1	-16.7	3.6
WAL781*	131819	F	14.1	-18.5	4.4	14.1	-17.6	3.7
WAL782	131814	F	13.1	-17.8	4.1	13.1	-17.0	3.5
WAL783*	131817	F	13.6	-19.3	5.1	13.7	-17.6	3.7
WAL784	131824	F	13.7	-18.0	4.6	13.8	-17.0	3.7
WAL785*	131820	F	14.1	-17.8	4.3	14.1	-16.7	3.4
WAL786*	131816	F	13.6	-19.4	6.0	13.9	-18.1	4.6
WAL787	131821	F	13.5	-19.5	5.4	13.6	-17.9	4.0
WAL788*	131818	F	13.3	-18.8	4.6	13.4	-17.8	3.8
WAL790*	131815	F	12.9	-18.2	4.3	13.0	-16.7	3.4
WAL791*	131799	M	11.9	-18.1	4.1	12.1	-17.3	3.6
WAL792*	131813	F	13.0	-18.5	4.5	13.3	-17.2	3.6
WAL793	131807	M	11.7	-18.5	4.9	12.4	-17.6	4.4
WAL794*	131796	M	11.8	-18.4	4.9	12.6	-17.7	4.3
WAL795*	131803	M	12.5	-18.3	4.1	12.6	-17.8	3.7
WAL796*	131808	M	11.7	-18.5	5.0	12.5	-17.7	4.5
WAL797*	131806	M	12.4	-18.1	5.3	13.3	-17.5	4.7
WAL798*	131800	M	11.6	-17.7	4.4	12.0	-16.6	3.7
WAL799*	131802	M	11.9	-17.8	5.1	12.0	-17.1	4.4
WAL800*	131809	M	11.6	-18.0	4.0	11.9	-17.4	3.5
WAL802*	131801	M	12.3	-18.1	4.4	12.8	-17.1	4.5
WAL803*	131798	F	13.1	-18.7	4.8	13.6	-17.7	3.9
WAL804	131805	M	12.3	-18.3	4.5	12.6	-17.1	3.6
WAL805*	131811	M	12.9	-18.2	5.4	13.3	-17.3	4.4
WAL806*	131797	M	11.7	-17.7	4.7	11.9	-17.2	4.1
WAL807*	131804	M	12.1	-18.0	4.6	12.0	-17.0	3.6
WAL808*	131810	M	13.4	-17.7	5.4	13.5	-17.2	4.6

Supplementary Dataset 4. Raw stable isotope data for walrus skin samples including animal ID, University of Alaska Museum specimen ID (UAM ID), sex, $\delta^{15}N$ (‰), $\delta^{13}C$ (‰), C:N, $\delta^{15}N_{lipid\text{-}free}$ (‰), $\delta^{13}C_{lipid\text{-}free}$ (‰), C:$N_{lipid\text{-}free}$. Asterisks next to IDs indicate individuals with all tissues (muscle, liver, skin, and bone collagen) used to evaluate tissue-specific isotope discrimination.

SKIN

ID	UAM ID	Sex	$\delta^{15}N$ (‰)	$\delta^{13}C$ (‰)	C:N	$\delta^{15}N_{lipid\text{-}free}$ (‰)	$\delta^{13}C_{lipid\text{-}free}$ (‰)	C:$N_{lipid\text{-}free}$
WAL137*	125312	M	14.4	-15.5	3.4	14.4	-14.7	2.8
WAL143	125318	M	14.0	-16.2	3.4	14.2	-14.8	2.9
WAL146*	129168	M	14.2	-14.4	3.2	14.3	-14.1	2.9
WAL234*	-	M	13.9	-17.3	3.7	14.3	-14.9	3.1
WAL235*	-	M	14.3	-15.2	3.3	14.4	-14.1	2.9
WAL238*	131795	M	14.2	-14.6	2.9	13.6	-14.2	3.0
WAL239*	-	M	13.6	-15.6	3.8	14.4	-13.9	3.0
WAL240*	-	M	15.0	-17.9	4.0	15.4	-15.4	3.1
WAL779*	-	F	15.7	-19.5	5.1	15.8	-15.4	3.2
WAL780*	131823	F	15.5	-15.6	3.2	15.2	-14.1	3.2
WAL781*	131819	F	15.2	-20.6	7.1	15.1	-14.7	3.2
WAL782	131814	F	14.3	-18.8	5.1	15.0	-14.9	3.0
WAL783*	131817	F	15.7	-18.6	4.1	16.0	-15.7	3.3
WAL785*	131820	F	15.4	-15.9	3.1	15.3	-15.1	3.2
WAL786*	131816	F	15.7	-16.4	3.3	15.7	-15.3	3.3
WAL788*	131818	F	14.8	-16.7	3.7	14.8	-14.0	3.2
WAL789	131822	F	15.0	-16.4	3.3	15.0	-15.6	3.0
WAL790*	131815	F	14.7	-18.8	5.2	15.3	-15.0	3.3
WAL791*	131799	M	14.8	-14.7	2.8	14.7	-14.5	2.9
WAL792*	131813	F	14.6	-16.3	3.2	14.9	-15.0	3.0
WAL794*	131796	M	14.0	-14.8	2.8	13.8	-14.7	3.0
WAL795*	131803	M	14.2	-20.1	6.6	14.3	-15.1	3.1
WAL796*	131808	M	14.3	-14.5	2.7	14.0	-14.4	3.0
WAL797*	131806	M	15.0	-14.9	2.8	15.1	-14.7	3.0
WAL798*	131800	M	14.6	-16.0	3.1	14.6	-15.2	3.1
WAL799*	131802	M	14.8	-14.7	2.8	14.8	-14.6	3.1
WAL800*	131809	M	14.5	-21.3	10.5	15.0	-15.1	3.3
WAL802*	131801	M	13.8	-17.6	4.3	13.7	-15.8	3.3
WAL803*	131798	F	14.8	-16.4	3.5	14.7	-15.1	3.0
WAL805*	131811	M	14.2	-16.3	3.4	14.2	-14.7	3.1
WAL806*	131797	M	14.6	-14.5	2.9	14.8	-14.4	3.1
WAL807*	131804	M	15.0	-20.9	8.8	15.3	-15.4	3.3
WAL808*	131810	M	15.3	-14.4	2.9	15.2	-14.2	3.1
WAL1002	125318	M	13.7	-15.7	3.2	13.6	-14.6	2.8
WAL1004	125325	M	14.0	-15.6	3.3	13.9	-14.5	3.0
